# In and out of Madagascar: Dispersal to Peripheral Islands, Insular Speciation and Diversification of Indian Ocean Daisy Trees (*Psiadia*, Asteraceae)

**DOI:** 10.1371/journal.pone.0042932

**Published:** 2012-08-10

**Authors:** Joeri S. Strijk, Richard D. Noyes, Dominique Strasberg, Corinne Cruaud, Fredéric Gavory, Mark W. Chase, Richard J. Abbott, Christophe Thébaud

**Affiliations:** 1 Key Laboratory of Tropical Forest Ecology, Xishuangbanna Tropical Botanical Garden, Chinese Academy of Sciences, Menglun, Mengla, Yunnan, People's Republic of China; 2 Laboratoire Evolution et Diversité Biologique, UMR CNRS 5174 – Université Paul Sabatier – ENFA, Toulouse, France; 3 Department of Biology, University of Central Arkansas, Conway, Arkansas, United States of America; 4 Université de la Réunion, UMR PVBMT, Faculté des Sciences et Technologies, Saint-Denis, La Réunion, France; 5 CEA, Institut de Génomique, Genoscope, Centre National de Séquençage, Evry, France; 6 Jodrell Laboratory, Royal Botanic Gardens, Kew, Richmond, Surrey, United Kingdom; 7 Mitchell Building, School of Biology, University of St. Andrews, St. Andrews, Fife, United Kingdom; University of California, Berkeley, United States of America

## Abstract

Madagascar is surrounded by archipelagos varying widely in origin, age and structure. Although small and geologically young, these archipelagos have accumulated disproportionate numbers of unique lineages in comparison to Madagascar, highlighting the role of waif-dispersal and rapid *in situ* diversification processes in generating endemic biodiversity. We reconstruct the evolutionary and biogeographical history of the genus *Psiadia* (Asteraceae), a plant genus with near equal numbers of species in Madagascar and surrounding islands. Analyzing patterns and processes of diversification, we explain species accumulation on peripheral islands and aim to offer new insights on the origin and potential causes for diversification in the Madagascar and Indian Ocean Islands biodiversity hotspot. Our results provide support for an African origin of the group, with strong support for non-monophyly. Colonization of the Mascarenes took place by two evolutionary distinct lineages from Madagascar, via two independent dispersal events, each unique for their spatial and temporal properties. Significant shifts in diversification rate followed regional expansion, resulting in co-occurring and phenotypically convergent species on high-elevation volcanic slopes. Like other endemic island lineages, *Psiadia* have been highly successful in dispersing to and radiating on isolated oceanic islands, typified by high habitat diversity and dynamic ecosystems fuelled by continued geological activity. Results stress the important biogeographical role for Rodrigues in serving as an outlying stepping stone from which regional colonization took place. We discuss how isolated volcanic islands contribute to regional diversity by generating substantial numbers of endemic species on short temporal scales. Factors pertaining to the mode and tempo of archipelago formation and its geographical isolation strongly govern evolutionary pathways available for species diversification, and the potential for successful diversification of dispersed lineages, therefore, appears highly dependent on the timing of arrival, as habitat and resource properties change dramatically over the course of oceanic island evolution.

## Introduction

The Madagascar and Indian Ocean Islands (MIOI) biodiversity hotspot is renowned for its high levels of species diversity and endemism [1]–[3], yet despite many hypotheses and a considerable literature, we still know relatively little about the mechanisms of species diversification within this region [1], [4], [5]. The break-up of East Gondwana (120–160 Ma) and the isolation of Madagascar and India have historically been attributed a major role in the assembly of the modern biota [6], [7], and until recently, Gondwanan vicariance was generally accepted as the most plausible explanation for the unique biota present on Madagascar (for a comprehensive discussion, see [8], [9]). However, an increasing number of studies show that the origin for many Madagascan groups post-dates the isolation of Madagascar [6], [9]–[15]. As such it appears more likely that present-day levels of diversity and endemism in Madagascar are due to a combination of divergence-by-vicariance and *in situ* diversification by post-break-up Cenozoic dispersers of mostly African origin [4], [9], [16], see also [17] and references therein). Upon reaching Madagascar, many colonizing lineages underwent extensive *in situ* speciation, whereas in some cases the founding taxa on the mainland became extinct leaving behind sets of ‘unique’ lineages in Madagascar [9], [18]–[22].

While Madagascar exhibits a distinct signature of evolution in isolation [9], it is also embedded in a wider geographical setting of oceanic, coralline and microcontinental islands that strongly vary in their origin, age and structure and may have played an important role in generating high species diversity and endemism at a regional scale. Some of these islands and others that are now submerged, have existed in the vicinity of Madagascar for long periods of time, up to 75 Ma [13], [23]. Although contributing little in terms of terrestrial area, archipelagos around Madagascar seem to have accumulated disproportionate numbers of species. This is particularly striking in some groups of organisms and on certain archipelagos. For example, in flowering plants, the Mascarene Archipelago, which comprises Réunion, Mauritius and Rodrigues, with a combined surface area of 4662 km^2^ (less than 1% of Madagascar), is inhabited by 960 species, approximately one tenth of the total number in Madagascar, of which 72% are endemic [23]. In birds, several lineages that are present on both Madagascar and surrounding islands contain similar or more species on the islands than on Madagascar itself [13], [14], [24], [25].

Madagascar has been a major source of colonizing lineages for surrounding islands with founders often, but not always, belonging to widespread and species-rich genera [23], [26]. The historical absence of direct connections to Madagascar or any other large island and continental region highlights the role of *waif* dispersal in generating present-day biodiversity on Indian Ocean islands surrounding Madagascar, and the potential for steppingstone dispersal and nested patterns of diversification within these archipelagos. Alternatively, it is also possible that such patterns arose from multiple unique colonization events followed by differentiation within islands with no or little *in situ* speciation. Furthermore, the high levels of endemism and recent origin of some islands (0.2–15 Ma) underscore the significance of high rates of evolutionary diversification in an insular setting, propelled by novel ecological and geographical opportunities [27], [28].

**Figure 1:**
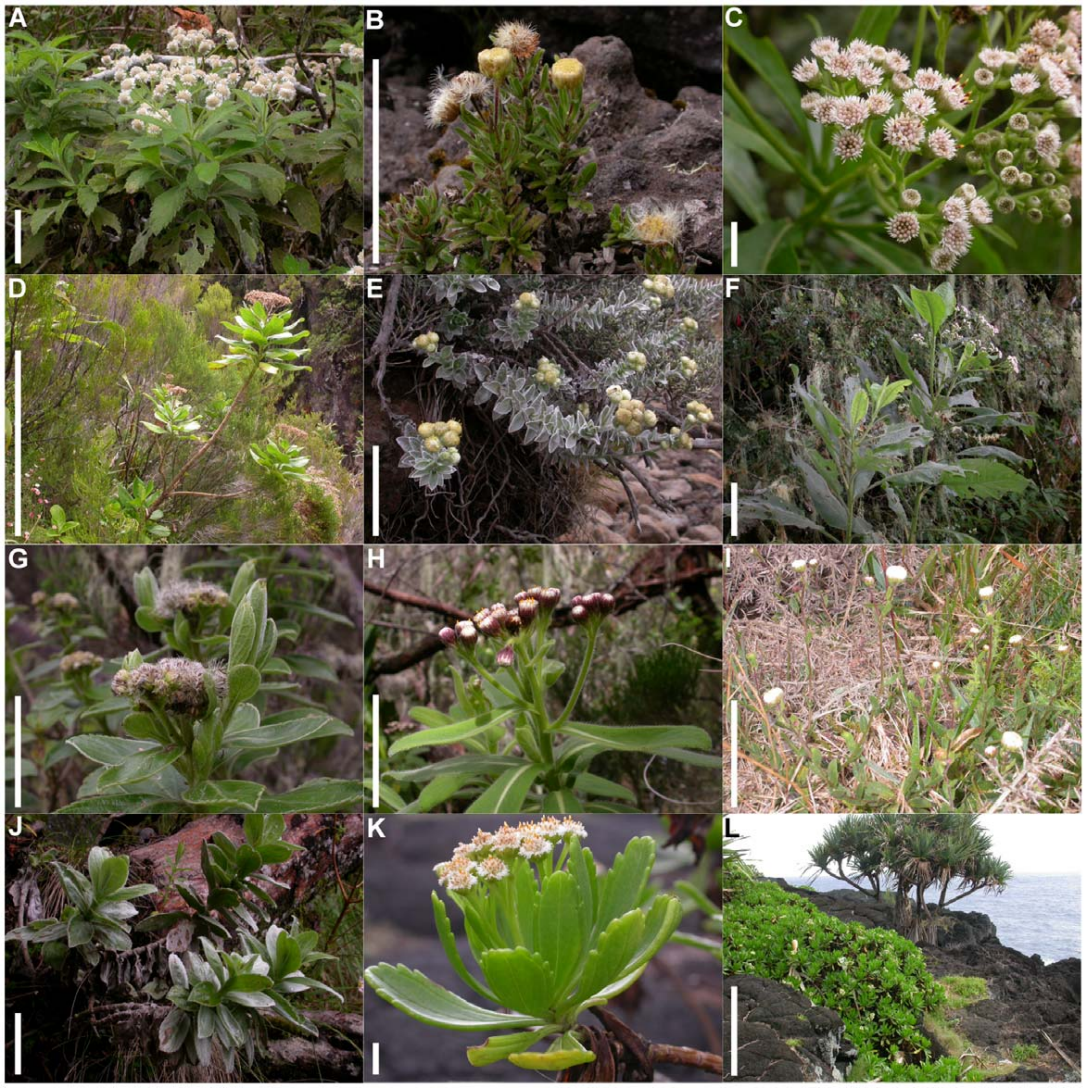
Phenotypic diversity and habitat of *Psiadia* on Réunion. *A: P. montana. B: P. callocephala. C: P. laurifolia. D: P. boivinii. E: P. argentea. F: P. insignis. G: P. melastomatoides. H: P. anchusifolia. I: P. aspera. J: P. salaziana. K: P. retusa.* L: Coastal habitat with *P. retusa.* Scale bars indicate 1 cm (C, K), 10cm (A, B, E, F, G, H, I, J) and 100cm (D, L). Photos by C.Thébaud.

**Figure 2:**
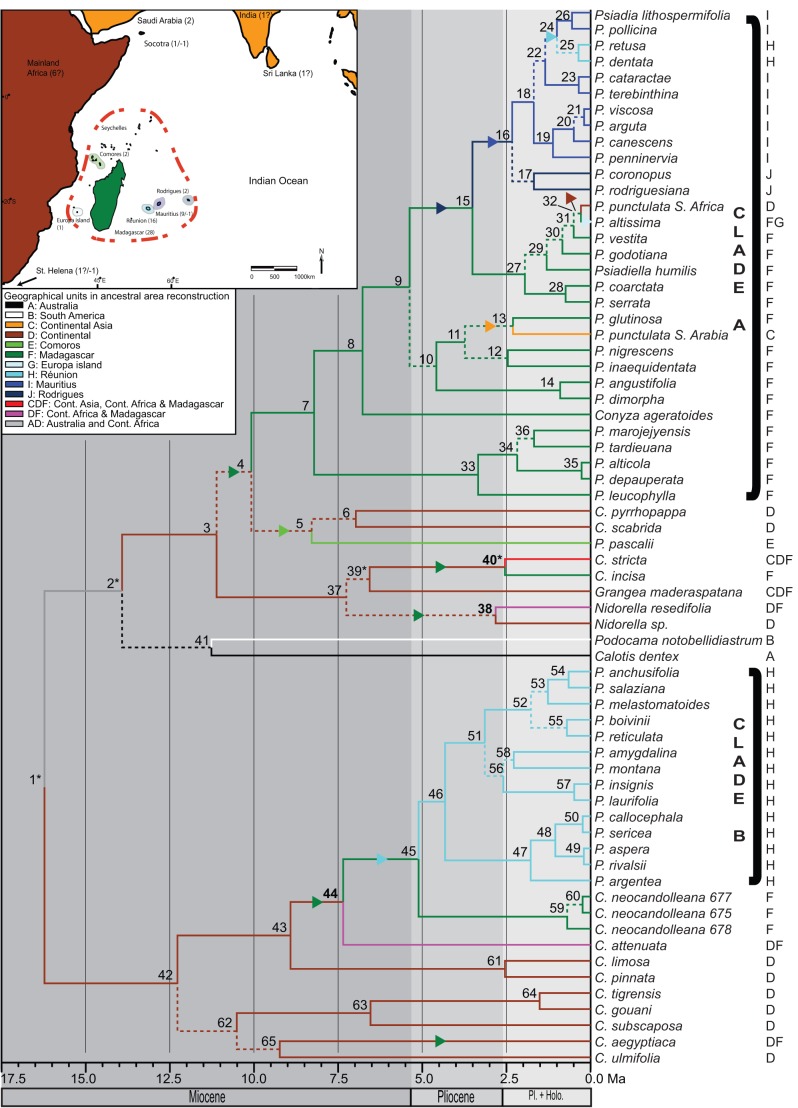
Ancestral area reconstruction using the dispersal-extinction-cladogenesis model. Inset map shows the Madagascar and Indian Ocean Islands biodiversity hotspot (dashed line) and the distribution and number of species of *Psiadia* (negative values indicate recorded extinctions of putative members of the genus). Contours of smaller islands have been enlarged and highlighted for clarity. Branch colors follow the designated geographic areas on the inset map. Bold node numbers indicate differences in optimal resolution between reconstruction using the dec-model and statistical Dispersal-Vicariance analyses. Asterisks highlight nodes for which multiple equally probable solutions were retrieved using Lagrange and/or S-DIVA. Arrows indicate dispersal events between designated regions. Node numbers refer to per-clade results listed in Table S3. Geographic coverage is indicated by capitals following taxon names. Broken branches lead to nodes with low posterior probabilities (<90) obtained in BEAST.

Understanding how the historical sequence of colonization and diversification shaped present-day biogeographic patterns of species diversity within the MIOI hotspot requires evaluating the spatial structure and timing of divergence events in component lineages at the scale of the entire region. In this study, we investigate how historical factors could explain present-day diversity and distribution of Indian Ocean daisy trees (*Psiadia* Jacq., Asteraceae), a phenotypically diverse (Figure 1), and species-rich group of plants with almost equal numbers of species in Madagascar and the Mascarene Archipelago. *Psiadia* contains 28 species in Madagascar and at least 30 species on surrounding islands, 27 of which are endemic to the Mascarene Archipelago (Figure 2, inset), with an estimated six species confined to the African mainland. *Psiadia* occur as (sub-)shrubs to small trees ranging in height from 10–20 cm for the smallest to over 10 m for the tallest species. Species occupy a diverse range of habitats from sea level up to 3000 m. Insular Asteraceae have provided many classic examples of extensive diversification in some of the most remote island systems in the world, and at least 27 genera occur throughout the islands of the Western Indian Ocean. As such, genera in this family provide us with an excellent opportunity to examine factors that could explain the origin and potential causes of species accumulation on islands peripheral to Madagascar and to understand the role of evolutionary radiations in generating the extraordinary levels of biodiversity in the MIOI hotspot.

Here we provide the first reconstruction of the evolutionary history of *Psiadia* in the Western Indian Ocean basin, using historical, geological, and plastid and nuclear DNA sequence data to infer the spatial and temporal patterns of colonization and speciation at the scale of the entire MIOI hotspot. The dissimilarity in species numbers of our study group found on the African mainland vis-à-vis Madagascar and the geologically young Indian Ocean Islands leads us to hypothesize the occurrence of shifts in rates of diversification following colonization events. With the majority of *Psiadia* concentrated in Madagascar and the likely recent origin of Mascarene species, the potential for changes in diversification rates, and the presence of rate bursts in particular, could serve to explain current high levels of endemism and species richness. We test for the presence of rate variation and significant rate shifts while attempting to place results in a wider ecological and biogeographical context. We perform ancestral area reconstruction to trace the origin of our study group and its movement throughout the western Indian Ocean basin. Finally, we examine whether there are general properties of diversification that are shared by species clusters on Indian Ocean islands and potential mechanisms that could be responsible for these patterns using distributional and ecological data.

We show that the genus *Psiadia* is polyphyletic and consists of two main species clusters, each having a unique history of dispersal and diversification in the Madagascar and Indian Ocean Islands biodiversity hotspot. One of these lineages is endemic to the island of Réunion with fourteen species. Over the course of its evolution, *Psiadia* experienced several statistically significant bursts of radiation following dispersal into new and isolated habitats. Throughout its range, the genus has followed a pattern of unidirectional colonization and dispersal, forming clusters of single island endemic species, following the appearance of new volcanic islands. Traces of intra-island diversification are most extensive on the geologically young and habitat-diverse Réunion, relative to that on older islands such as Mauritius and Rodrigues, which appear to have entered the final phases in the geological sequence of volcanic islands (i.e. erosion, decay and submergence) and now contain a reduced set of habitat types and environmental conditions compared with Réunion.

## Methods

### Taxon sampling

Specimens from sites in Madagascar, the Mascarenes and the Comoros were dried in silica during extensive fieldwork (vouchers stored at the University of Toulouse DNA bank and the University of Réunion herbarium at St. Denis). All samples were collected by specialists on *Psiadia* and related genera (CT, RN, RJA, DS). Of the more than 65 described species within the region, 45 were included in the study. Additional samples were obtained from DNA banks in Kew (UK), Missouri (USA) and Kirstenbosch (South Africa). Several historically documented species occur outside the main range of the genus (Figure 2, inset). Multiple accessions were included for a widespread species (*P. punctulata*) and the Madagascan monotypic *Psiadiella humilis*, as we suspected it should be included in *Psiadia*.

To evaluate the monophyly of *Psiadia*, we used a range of outgroups from within the Astereae. We found that several genera occur in the region which, using morphology alone, have often been mistakenly identified as *Psiadia* and vice versa (J.S. Strijk, unpublished data). Of these, *Conyza* is noteworthy due to its size (∼50spp.) and questionable circumscription of its species. We were unable to include samples of all currently recognized species, but included fourteen species to assess the gross relationship between *Psiadia* and regionally endemic *Conyza*.

Previous phylogenetic studies within Astereae that rooted within the tribe gave conflicting ingroup relationships [29]–[31] and we therefore followed Cross [32] and included several members of the sister-tribe Anthemidae [33]. To enable estimates of divergence times we added representatives from several other tribes (Tageteae, Heliantheae and Gnaphalieae), using sequences deposited in GenBank.

### DNA sequencing and alignment

We generated new sequence data for four molecular markers (*acc*D, *rpo*B, *psb*A*-trn*H and nrITS; [34]–[36]) from 51 species. Voucher and GenBank accession data are provided in Table S1. Details of primer pairs, DNA isolation, purification and PCR conditions can be found in Tables S2 and S3. DNA sequence data were assembled and edited with Sequencher v4.7 (Gene Codes, Ann Arbor, Michigan). Alignments were constructed manually using SE-AL v2.1a11 [37]. Ambiguously aligned sections were excluded from the analyses and indels were coded using the simple coding method implemented in GAPCODER [38].

### Phylogenetic Analyses

We conducted initial phylogenetic analyses on a combined dataset using MrBayes v3.1.2 [39], [40] with model partitioning on the Vital-IT cluster of the Swiss Institute of Bioinformatics (www.vital-it.ch/). Optimal model selection for the four partitions was performed previously using the Akaike Information Criterion (AIC) in MODELTEST v3.7 [41]. Indel characters were treated as fifth partition, with the restriction site model and ‘variable’ option in effect. Metropolis-Coupled Markov Chain Monte Carlo (*MC^3^*) sampling was performed with four chains running for 20*10^6^ generations, sampling every 1000^th^ generation and discarding the first 50% of sampled trees as burnin. In addition, we analyzed our data using PHYCAS [42], [43] to ascertain the stability of backbone nodes with lower support values. PHYCAS uses a reversible-jump MCMC algorithm to allow trees with polytomies or very short and poorly supported branches, and fully resolved, well supported tree topologies to compete during a Bayesian analysis. This allows for detection of inflated support values obtained in ML and standard BI analyses of rapidly radiated groups, and allows assessment of unresolved alternatives. We implemented a polytomy prior of *e*, and ran PHYCAS twice for 100,000 cycles (1 cycle in PHYCAS corresponds to roughly 100 iterations in MrBayes), sampling every 10 cycles and setting a burnin value of 2000.

Finally, we performed likelihood tests on alternative phylogenetic hypotheses (monophyly of ((*Psiadia*, *Psiadiella*), (*Conyza*)); (*Psiadia*, *Psiadiella*); (*Psiadia*, *Psiadiella*, *Conyza*); (*Psiadia*) and (*Conyza*). Start up topologies were obtained using constrained analyses in RAxML v7.2.8 HPC-PTHREADS [44], [45], using optimized model settings, data partitioning and assessing support with 1000 bootstrap replicates. Per-site likelihood values were estimated in PAUP 4.0b10 [46]. We employed Consel [47] to test alternative hypotheses using the Approximate Unbiased (AU) and Weighted Shimodaira-Hasegawa (WSH) [48] tests and Shimodaira-Hasegawa (SH) [49] test.

### Estimation of Divergence Times

We estimated rates and divergence times with penalized likelihood (PL) implemented in R8s [50]–[52], using the TN algorithm. We applied all calibration constraints (except the root which was fixed as required in R8s) and used the MrBayes consensus tree as a starting tree (prior settings in Table S4). A cross-validation procedure to determine the rate smoothing parameter *λ*, with the additive penalty setting was performed in PL. Uncertainty in divergence-age estimates for nodes of interest was assessed by bootstrap resampling of the original dataset on a fixed topology in TREEFINDER [53]. Resulting trees were processed in R8s to summarize variation in branch length estimates using the *profile* command.

Bayesian analysis using the uncorrelated lognormal (UCLN) model [54] was performed in BEAST [55]. Using model partitioning following [56] and selecting a Yule prior for the branching process we analyzed two profiles with (prior settings in Table S4B) with the ultrametric tree obtained in R8s as starting tree. After conducting several short runs to adjust operator settings, two independent Markov Chain Monte Carlo (MCMC) runs were conducted for 20*10^6^ iterations, of which 5*10^6^ iterations (25%) were treated as burnin. Increasing the chain length to 50*10^6^ iterations had no significant impact on divergence age estimates (data not shown). Mixing of the chains and convergence of the runs were checked in TRACER [57]. Converged runs were combined with LOGCOMBINER [58] and the maximum clade credibility (MCC) tree was constructed using TREE-ANNOTATOR [59]. The XML-file implemented in BEAST is available from the corresponding author upon request.

### Fossil constraints and calibration

Asteraceae have been the subject of intensive studies using phylogenetic and morphological data, but the origin and diversification of major clades have remained enigmatic owing to their generally poor early fossil record [60]–[65]. Most fossil data consist of pollen deposits and occasionally fruits [66], [67]. Although still uncommon in the Eocene, Asteraceae pollen increases in abundance from the Oligocene and Miocene onward.

Previously published age estimates for Mascarene Islands have cited only geological ranges based on potassium-argon and stratigraphic dating. It is estimated that the Mascarene volcanic hotspot first began to generate Mauritius some ∼8–10 Ma [68], [69]. While Mauritius drifted north-eastward, Réunion surfaced between ∼2–5 Ma. The small island of Rodrigues is generally thought to be the youngest (∼1.5 Ma), but age estimates are based on a limited and much cited geological study [69]–[71]. New geological and phylogenetic evidence consistently suggests a more ancient origin of Rodrigues [70], [72], [73].

To take into account existing uncertainties on exact geological island age estimates and to avoid circularity we chose not to calibrate our tree directly using published island age estimates [74]. Instead we used a set of external fossil dates for calibration, allowing us to evaluate the biogeographic and evolutionary scenario of island evolution pertaining to our study group, while being conscious to avoid the introduction of weakly supported and potentially conflicting age constraints. Although we attempted to rely only on original fossil data, we have included two secondary calibrations in our analyses. The use of secondary calibration data obtained in previous studies can be problematic due to the build-up of error introduced by the original analysis. Regrettably, no suitable fossils for direct calibrations exist within our study group, and we therefore have attempted to use a balanced approach to calibrate our analyses using a wide-outgroup approach (involving both primary and secondary calibrations), which, in the absence of abundant reliable fossil data, is a reasonable alternative [75]. Throughout this study we used the division and bounds of absolute geological ages from Gradstein [76].

We applied multiple calibrations spread throughout our tree and used a combination of calibration priors and prior types to test for uncertainty in published fossil dates and error in secondary calibrations. Our initial analysis (Beast-1 – Table S4B) was performed using only uniform priors with wide bounds to explore the space in which the posterior estimates respond, and follow up analyses (Beast-2 – Table S4B) were performed using alternate settings discussed next.

Previous studies have provided a number of age estimates on the historical origin of the family and the subfamily Asteroideae. In a study by Bremer using substitution rates and fossil pollen data [77], the origin of the family was inferred to date back to at least 38 Ma (Mid-Late Eocene, sensu [76]). Fossil pollen samples attributed to Asteraceae have been found on all southern hemispheric continents and the family is believed to have originated in (southern) South America following a split from Calyceraceae [78]–[81]. More recent studies on outstanding fossil material for Asteraceae found in Eocene deposits of northwestern Patagonia dated to 47.5 Ma [82]–[84] push back the age of origin and are more or less in line with previous age estimates for the whole family using paleogeographic reconstruction [79](and references therein).

**Figure 3:**
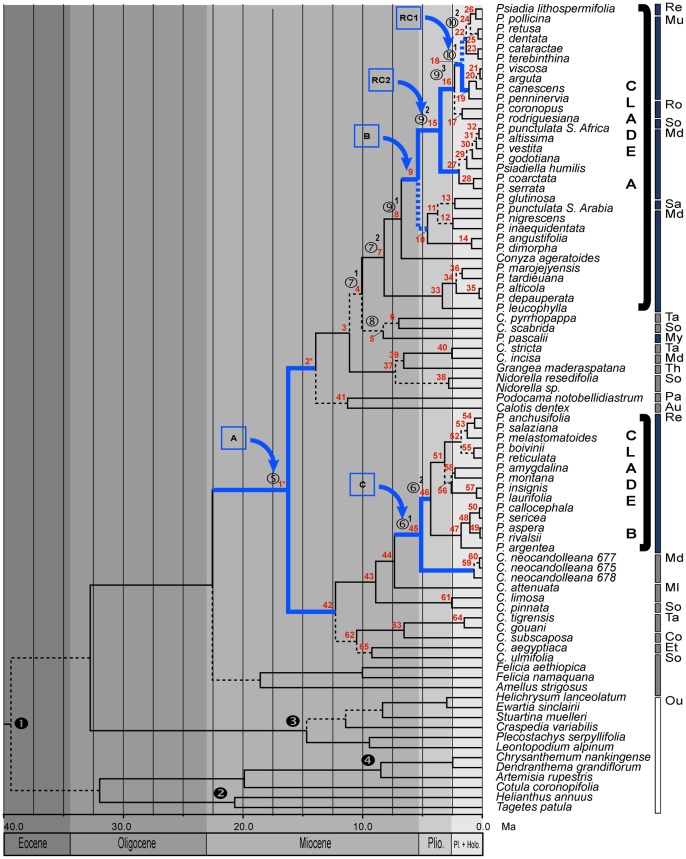
Chronogram for *Psiadia* s.l. and results of diversification rate shift analyses. Broken branches lead to nodes with low posterior probabilities (<90) obtained in BEAST. Arrows indicate rate shifts associated with results obtained using SYMMETREE (rate shift box A, B & C: Δ1, Δ2 and SG) and the Relative Cladogenesis test (RC1 & RC2). Numbered nodes 1–4 indicate calibrated nodes. Numbered Nodes 5–10 refer to key events in the evolutionary history of *Psiadia*, as discussed in text. Bars: blue, *Psiadia* clade A & B; grey, other taxa contained with *Psiadia*-clades; white, outgroup taxa. Abbreviations: Re (Réunion), Mu (Mauritius), Ro (Rodrigues), Md (Madagascar), So (South Africa), Sa (Saudi Arabia), Ta (Tanzania), My (Mayotte), Th (Thailand), Pa (Paraguay), Au (Australia), Ml (Malawi), Co (Congo-Kinshasa), Et (Ethiopia), Ou (Outgroups).

The root node of our tree corresponds to the crown node of the subfamily Asteroideae, and we used results obtained in a previous study based on fossil data using NPRS and substitution rates of *ndh*F [63], ranging from 26–29 Ma (NPRS) to 35–39 Ma (substitution rates). We applied a normal prior and used the midpoint of these age estimates with a 95% confidence interval of 14.1–50.9 Ma and a mean of 32.5 Ma (node 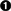 in Figure 3).

Age estimates were previously published for South African Gnaphalieae [85] using a combination of calibration points and types. We collected sequences used in this study from GenBank and set priors for two nodes following the authors' methodology and results. For the node containing Gnaphalieae we applied a normal prior with a mean of 20.0 Ma (95% CI: 12.0–29.3 Ma), placing it in the Early Miocene (node 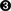 in Figure 3).

In addition, we used a previously published age estimate [66], [86] for the earliest *Ambrosia*-type pollen (Heliantheae) to calibrate the node connecting *Helianthus annuus* and *Tagetes patula* (Tageteae). Fossil pollen data were dated to the Eocene-Oligocene border (25–35 Ma) and we applied a lognormal prior with a mean of 22.3 Ma (95% CI: 16.9–44.1 Ma; following [85]; node 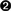 in Figure 3).

Finally, the fossil record for *Artemisia* and its implications for historical and phylogenetic reconstruction were discussed by Wang [87]. The abundance of fossil pollen confidently assigned to *Artemisia* and the outcome of phytogeographical reconstructions suggest that the genus did not thrive until the Middle to Late Miocene, although it is thought to have been present in low densities for as far back as the Late Paleogene [81], [88]. The geographical coverage of fossil finds is extensive with records from the Late Oligocene (central Europe) and Early and Middle Miocene (western and eastern North America, respectively) [66]. To accommodate the uncertainty in the timing of origin we assigned a lognormal prior allowing for a wider sloping interval with a mean of 16.98 Ma (95% CI: 5.44–28.52 Ma; node 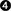 in Figure 3).

### Tempo of Diversification

The γ-statistic [89] of the R-package LASER [90] was calculated, using the ultrametric trees generated by BEAST for our two ingroups, to explore whether speciation events have accelerated (γ>0) or decelerated (γ<0) towards the present as opposed to expectations under a constant rate (CR) model (γ = 0). The null hypothesis of constant birth and death rates (*alpha*  = 0.05, one-tailed) can be rejected if γ<−1.645. Undersampling can have a negative impact on the estimation of γ and to explore the effects of incomplete taxon sampling on our estimate of γ, we used a Monte Carlo constant rate test (*mccrTest*; R-package LASER [90]) in the analyses of *Psiadia* (31 spp. sampled out of 51). We calculated the adjusted γ by simulating 10,000 trees under a pure birth model for the number of extant taxa and by randomly pruning the missing number of taxa from the constructed trees.

Lineage-Through-Time plots were constructed in GENIE v3.0 [91] using the BEAST chronogram. We evaluated the effects of incomplete taxon sampling on the slope of our empirical LTT-curves by generating 1000 simulated trees of the extant number of taxa in both groups using a constant rates model in PHYLOGEN v1.1 [92]. We proceeded by randomly pruning the unsampled number of taxa from these trees and rescaling the branch lengths using TREE-EDIT v1.0 [93] to set the age of the root nodes to the crown node age estimates obtained in BEAST. Trees were used to construct mean LTT-curves and 95% confidence intervals for comparison with the empirical curves.

### Diversification Rates and Rate Shifts

Topological and temporal methods were used to study diversification rates and rate shifts in our study group. The topological program SYMMETREE v1.1 [94] was employed to conduct whole-tree tests for diversification rate-variation, using the M-statistics (*M_r,_ M_Σ_*, M_Σ_*, *M_Π_* and M_Π_*) to test for the presence of significant rate shifts [95], [96], the Slowinski-Guyer (SG) statistic [97], [98] and *Δ_1_*/*Δ_2_* likelihood rate shift statistics to locate shifts and the *B_1_* and *I_c_* statistics to assess tree imbalance [99]–[101]. We used the consensus tree obtained in MrBayes, the pruned ingroup clades A and B and the MCC chronogram of our BEAST analyses. The Relative Cladogenesis (RC) test was performed using GEIGER [102]. Analyses were conducted both for the whole tree as well as for pruned ingroup clades to rule out bias (‘trickle-down-effect’ [96]).

Changes in diversification rates were explored using the temporal method birth-death likelihood (BDL) available in LASER [103]. BDL is the only method currently available that can differentiate between a temporal increase in diversification rates and a rate-constant model in which extinction is greater than zero, thus avoiding the “pull of the present”-effect which can lead to incorrectly inferring an increase in the net diversification rate [89], [104]. We pruned outgroups and intermittent taxa from the BEAST chronogram, and set the root of our two ingroup trees to 4.32 Ma (B) and 8.21 Ma (A). The presence of rate variation in our two ingroups was assessed by evaluating the fit of two rate-constant and four rate-flexible diversification models. The best model was selected by calculating the difference in Akaike Information Criterion scores (ΔAIC_rc_) between the best rate constant and rate-flexible model [103]. We assessed significance by simulating branching times under a pure birth model for 5000 trees (*yuleSim*), given the same number of species as present in our phylogeny and using the estimate of the speciation rate obtained under the pure-birth model in BDL.

### Ancestral Area Reconstruction

Two approaches were used to reconstruct the evolution of ancestral ranges of *Psiadia*. First, we used a likelihood approach with the package LAGRANGE v. 20110117 [105] implementing a dispersal-extinction-cladogenesis model (*dec*-model – latest version and online *Python* script configurator at http://www.reelab.net/lagrange/configurator/index). The MCC tree obtained with BEAST was used as the input tree.

Secondly, we employed the software package RASP 2.0 [106] to run Statistical Dispersal-Vicariance Analysis (S-DIVA, now contained in RASP) [107]. Reconstructions were performed independently on 1,000 trees chosen randomly from the post burnin BEAST trees-file and projected results onto the final BEAST MCC tree. We identified eight areas covering the extant distribution of *Psiadia* species based on the geological history of the western Indian ocean and patterns of endemism in the genus (see Figure 2, inset). Regional assignment was performed based on floristic accounts [108]–[110], the most recent publications including distribution data [111]–[113] and expert knowledge on species ranges (CT, RN, RJA, DS).

## Results

### Phylogenetic analyses

All data partitions were analyzed separately and showed no strongly supported topological conflicts (bootstrap percentages (BP) ≥80 or posterior probability (PP) ≥0.95). We therefore used a combined dataset for further analysis. First, the full set of accessions with multiple entries for most species was examined, but this was reduced to a smaller set of accessions in subsequent analyses (Table S1). Results presented here are for analyses conducted using a combined dataset comprising the following sequences, accessions and characters: 1) *acc*D: 39 accessions, 296 characters; 2) *rpo*B: 38 acc., 427 chars; 3) *psb*A*-trn*H: 47 acc., 404 chars; and 4) ITS; 83 acc., 783 chars.). Using GapCoder, we added an additional 83 characters (*psb*A*-trn*H: 15; ITS: 68). Phylogenetic analyses using maximum likelihood (RAxML) and Bayesian (MrBayes v3.1.2) approaches yielded highly congruent topologies and rendered *Psiadia* non-monophyletic within monophyletic Astereae (Figure S1). Differences existed between likelihood and Bayesian support values and we employed PHYCAS to determine whether results obtained with MrBayes were unjustified or inflated. Results indicate that, even when topologies containing polytomies are considered, the nodes forming the backbone of the phylogeny remain resolved with mostly moderate (4 nodes) to high (10 nodes) support (Figure S1).

Two independent clades containing *Psiadia* species were recognized (Figures 2 and 3). Clade A comprised accessions of *P. punctulata* from southern Africa and Arabia, all species from Madagascar, Rodrigues, Mauritius, and two species from Réunion (BP/PP/PH:75/1/1), while clade B contained 14 of the 16 species from Réunion (BP/PP/PH:100/1/1). Also included within clade A were *Psiadiella humilis* and *Conyza ageratoides* (both endemic to Madagascar) These appeared as sister to or in subclades dominated by Madagascan taxa. The two accessions of *Psiadia punctulata* from Southern Africa and Saudi Arabia were placed in different sub-clades of clade A indicating that this taxon should no longer be treated as a single lineage with a very wide geographic distribution, but rather as two (at least) distinct species. Clades A and B were topologically separated from each other by nearly all other Astereae included in our study (except *Felicia* spp. and *Amellus strigosus*) underscoring their different evolutionary origins and history.

The fourteen *Conyza* species included did not constitute a monophyletic group, but rather formed several smaller, mostly weakly supported clades of 2–5 species throughout the tree (Figure 3 and Figure S1), indicating that this genus is polyphyletic, with Madagascan *C. neocandolleana* sister to Réunion *Psiadia* in clade B. Likelihood testing of alternative topologies using CONSEL strongly rejected hypotheses of monophyletic *Psiadia* (*P*<< 0.001) and *Conyza* (*P*<<0.001), and different combinations of these with/without *Psiadiella* (*P*<<0.001) (Approximate Unbiased (AU), Weighted Shimodaira-Hasegawa (WSH) and Shimodaira-Hasegawa (SH) tests).

### Estimation of Divergence Times

We used the consensus tree obtained with MrBayes as the input tree for divergence time estimation in R8s. Results of PL-bootstrapping on divergence estimates that incorporated only the compulsory fixed root node (32.5 Ma) varied little from analyses using the full set of node calibrations (Table S4A) for our nodes of interest. Overall, results suggest a Late Miocene origin (7.57/7.98 Ma) for the age of the most recent common Madagascan ancestor of *C. neocandolleana* and the *Psiadia* of clade B. Lineage splitting occurred soon after colonization of Réunion with the two major early lineages in clade B appearing some 6.6/7.03 Ma. The origin of species in clade A is resolved as older, with root node (14.48/15.05 Ma) and crown node age estimates (12.98/13.5 Ma) placing it in the Middle Miocene. The origin of the Mascarene *Psiadia* in clade A appears to lie in the Mid-Late Miocene (root node age estimate: (9.45/9.97 Ma), with the radiation on the islands taking place closely before the start of the Pliocene (crown node age estimate: (5.46/5.88 Ma).

We rejected the assumption of a molecular clock for the combined data set (*p*<0.0000001, df  = 81), and using our calibration scheme (Table S4B), we estimated divergence times for all nodes using a Bayesian relaxed molecular clock model in BEAST. Adequate mixing of the chains and convergence of the runs was checked in TRACER, showing effective sample size (ESS) values for all parameters to exceed 400. Maximum Clade Credibility trees (MCCs) obtained were largely congruent with topologies obtained in maximum likelihood and Bayesian analyses. Although age estimates for some nodes between the two calibration sets show considerable variation (in particular for nodes where uniform priors were employed), estimates for nodes of primary interest in this study varied little between calibration schemes (*µ* = 1.91, *σ* = 1.04).

**Figure 4:**
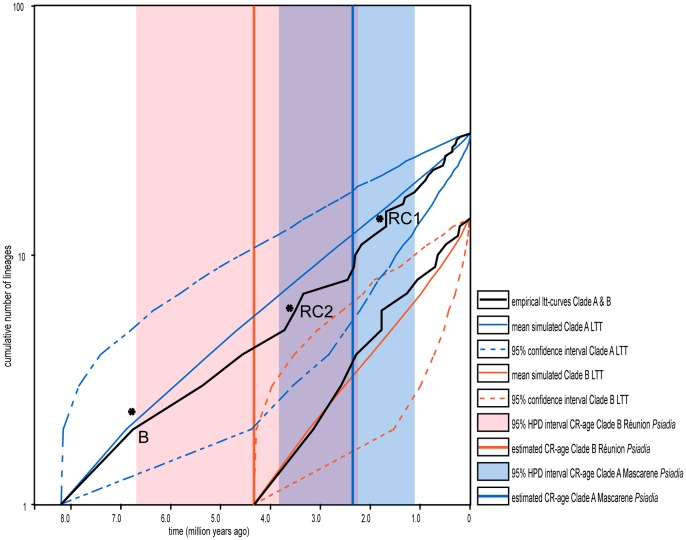
Lineage through time curves (with 95% confidence intervals) for clades A and B. Black lines: individual empirical curves from the BEAST analysis. Blue (clade A) and red (clade B) lines demarcate 95% confidence intervals generated using a constant rates model. Included rate shifts (*) and 95% HPD intervals shown as described in text and Figure 3.

Node-age estimates obtained using BEAST differ from values obtained with R8s, with most Bayesian estimates being younger. The cause for the differences in age estimates may lie in the fact that BEAST uses a relaxed clock approach to derive the posterior distribution of rates and times, and allows for the specification of different types of calibration distributions to model calibration uncertainty [114]. In contrast, R8s only employs user-supplied minimum and maximum age constraints as starting priors to construct node age estimates. Support for the topology and nodes of interest is high with the exception of the root node of clade A (PP: 0.52). A normal prior on the root node for Asteroideae resulted in an age estimate of 39.4 Ma (95% HPD (Highest Posterior Density): 28.5–51 Ma), which is older than the estimate by Kim [63], but confidence intervals are largely overlapping. We obtained an estimate of 4.3 Ma (95% HPD: 2.3–6.7 Ma) for the colonization of Réunion from Madagascar by the ancestor of clade B, which produced two intra-island radiations soon after colonization (5.11 Ma; 95% HPD: 2.6–7.8 Ma). In comparison, age estimates for clade A are older, with root node (10.1 Ma; 95% HPD: 5.9–4.5) and crown node (8.2 Ma; 95% HPD: 4.8–12.2) estimates placed well in the Late Miocene. Estimates and confidence intervals indicate that by the time species of clade A *Psiadia* from Madagascar reached Réunion via Rodrigues and Mauritius (1.0 Ma; (95% HPD: 0.3–1.9)), the ancestor of clade B had already diversified on Réunion into the main lineages that gave rise to modern-day species (Figure 4).

### Tempo of Diversification

Because phylogenetic analysis indicated that *Psiadia* is non-monophyletic, with a clear separation into two large clades, we proceeded to examine the two clades in a comparative analysis of diversification patterns and rates. Using floras, online resources and expert knowledge of the genus we assessed the currently described number of species of clade A to be 51 of which our sample included 31 (∼61%) covering the main distribution. The Mascarene Islands have been particularly well surveyed, and this study included all known species of clade B (14), as well as the described but previously unpublished *P. reticulata*. Constant rate tests resulted in a positive γ-value for *Psiadia* in clade A (γ = 0.58) indicative of the majority of cladogenesis distributed towards the tips of the phylogeny. A negative γ-value was obtained for Réunion *Psiadia* in clade B (γ = −0.09) suggesting a pattern of early branching and a subsequent marked slowdown in rate of cladogenesis. Taking into account the effect of undersampling on our estimate of γ using LASER, we found significant evidence to reject the constant rate-model (γ = −1.97) for clade A, but failed to reject the constant diversification model in the case of clade B. Lineage-through-time (LTT) plots, constructed in GENIE, reflect results of Constant Rate (CR) and Monte Carlo Constant Rate (MCCR) testing (Figure 4).

The curve for clade A, beginning ∼8.21 Ma, illustrates long periods of relatively stable rates of diversification followed by several upturns of short duration of rates during the Pliocene. The curve for clade B on Réunion shows an initially steeper curve with a slightly declining trend towards the present after a single sharp increase approximately ∼1.5 Ma. We combined LTTs of the two clades in Figure 4 to visualize the extent of their historical co-occurrence on the Mascarene Islands. We found that diversification rates in clade A significantly increased since the late Miocene, whereas diversification rates in clade B have been near constant with a tendency to slow down after the Pleistocene.

### Variation in Diversification Rates and Rate Shift Analyses

Results from the topological method implemented in SYMMETREE using whole-tree tests for rate-variation (M-statistics: *M_r,_ M_Σ_*, M_Σ_*, *M_Π_* and M_Π_* – presence of significant rate shifts; *B_1_* and *I_c_* statistics to assess tree imbalance) confirm the presence of both tree imbalance and significant variation in diversification rates between lineages at intermediate levels (Tables 1 and 2 (results for complete tree testing)). When restricted to ingroup clades only, support for variation was weak to absent, leading us to conclude that rate variation between lineages is mostly due to the presence of non-ingroup taxa (e.g. *Conyza*, *Nidorella*). Since taxon sampling for these groups was incomplete, detected variation and shifts at levels above our ingroup clades are not considered here.

**Table 1:** Results of relaxed Bayesian dating and diversification rate shift analyses for selected nodes.

*Node numbers*	Node description	Mean divergence estimate	95% HPD (BEAST)	PP (BEAST)	Diversification rate shift
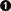	Asteroideae crown (root)	39.4	(28.5–51.03)	1	-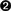	*Tagetes*-Helianthus split	20.7	(18.12–23.82)	1	-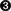	Gnaphalieae node	14.69	(8.67–20.48)	1	-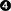	*Artemisia* root age	8.48	(5.81–12.19)	1	-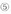	MRCA clade A and B	16.23	(9.93–23.45)	1	Shift box A:
					*Δ_1_* = 0.052
					*Δ_2_* = 0.061
					SG = 0.050
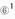	Clade B root age	5.11	(2.56–7.8)	1	Shift box C:
					*Δ_1_* = 0.082
					*Δ_2_* = 0.103
					SG = 0.285
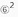	Clade B crown age	4.32	(2.25–6.69)	0.92	-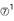	Clade A root age	10.08	(5.86–14.48)	0.52	-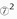	Clade A crown age	8.21	(4.76–12.15)	0.98	-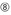	Split between *Psiadia pascalii – Conyza pyrrhopappa & C. scabrida*	8.29	(4.34–12.57)	0.84	-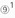	Split between lianescent *Conyza ageratoides and majority of clade A Psiadia*	6.77	(4.76–12.15)	0.99	Shift box B:
					*Δ_1_* = 0.047
					*Δ_2_* = 0.060
					SG = 0.178
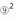	Clade A *Psiadia* Mascarenes root age	3.51	(1.76–5.58)	1	RC2: 0.033
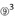	Clade A *Psiadia* Mascarenes crown age	2.33	(1.1–3.83)	1	-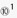	Crown node estimate of Mauritian and**Réunion *Psiadia*	1.69	(0.72–2.72)	0.89	RC1: 0.044
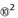	Root node estimate of split between Mauritian and Réunion *Psiadia*	1.00	(0.28–1.86)	0.33	-

Notes: Values in Ma. Node numbers refer to symbols used in Figure 3. Abbreviations: HPD – Highest Posterior Density interval; PP – Posterior Probability; MRCA – Most Recent Common Ancestor; SG – Slowinski-Guyer; RC – Relative Cladogenesis.

**Table 2:** ERM nodal probabilities obtained in whole-tree tests for diversification rate-variation using SYMMETREE.

Origin of tree	# taxa	*Frequentiles*	*B_1_*	*M_Σ_*	M_Σ*_	*M_Π_*	M_Π*_	*I_c_*
**MrBayes 50% mj.r. consensus**	83	*0.025*	0.009824	0.000093	0.000009	0.000038	0.000061	0.00033
	83	*0.975*	0.29749	0.01458	0.001702	0.002581	0.000932	0.001804
**BEAST chronogram**	81	*-*	0.93055	0.191548	0.011834	0.057509	0.00618	0.003049
**Pr. clade A**	31	*0.025*	0.025175	0.01838	0.007575	0.007569	0.004426	0.007363
		*0.975*	0.099621	0.095531	0.131621	0.143871	0.205166	0.298433
**Pr. clade B**	14	*0.025*	0.101303	0.0577637	0.113537	0.0976611	0.171532	0.240453
		*0.975*	0.952141	0.935058	0.935197	0.929431	0.933223	0.97365

Notes: Tests were conducted on the consensus tree obtained with MrBayes and the two separate pruned ingroup clades of clade A and B, and the MCC chronogram obtained with BEAST.

Using the Slowinski-Guyer, *Δ_1_* and *Δ_2_* likelihood rate shift statistics, we obtained the following *p*-values for a rate shift deep in the tree (*Δ_1_* = 0.052; *Δ_2_* = 0.061; SG = 0.050; box A, Table 1 and Figure 3). A rate shift along the branch leading to the majority of Madagascan and Mascarene *Psiadia* in clade A was inferred, following the split from *C. ageratoides* (*Δ_1_* = 0.047; *Δ_2_* = 0.060; SG = 0.178; box B, Table 1 and Figure 3). For the node supporting the onset of diversification in clade B we obtained the following *p*-values (*Δ_1_* = 0.082; *Δ_2_* = 0.103; SG = 0.285; box C, Table 1 and Figure 3). Results from the RC-test resulted in two significant rate shifts in clade A (RC1 = 0.044; RC2 = 0.033; Table 1 and Figure 3), corresponding to the split between Mauritian and Réunion *Psiadia* and between Madagascan and Mascarene *Psiadia*, respectively (Figures 3 and 4).

Results from the birth-death likelihood analysis (BDL) of ingroup trees are congruent with results from CR and MCCR tests (Table 3). The model best fitting the data from the clade B chronogram was the rate constant pure birth model, with a maximum likelihood estimate (*mle*) of speciation rate of 0.484 speciation events per million years. BDL identified the rate variable Yule-3-rate model as the best fit for the clade A chronogram, finding an *mle* of 0.413 events per million years (ΔAIC_rc_ = 3.618). A sharp shift in diversification rate was inferred at 1.69 Ma, corresponding with the timing of the first lineage splitting on Mauritius. Simulations, however, showed that the better score of the Yule-3-rate model, as opposed to the pure birth model, were non-significant (*p* = 0.061), most likely due to model overfitting (ΔAIC_rc observed_  = 3.62 < ΔAIC_rc critical_  = 4.07). Incorporating undersampling (31 out of 51 extant taxa) also did not lead us to reject the null hypothesis of a rate constant model of evolution (*p* = 0.071).

**Table 3:** Results of birth-death likelihood (BDL) analyses of lineage diversification.

Clade A											
Model	LH	r1	r2	r3	a	xp	k	st1	st2	AIC	ΔAIC_rc_
***Pb***	19.997	0.413								−37.993	3.618
***Bd***	20.199	0.320			0.357					−36.397	5.214
***DDX***	20.141	0.297				−0.130				−36.282	5.329
***DDL***	19.997	0.413					516353.7			−35.993	5.618
***Yule2rate***	20.697	0.291	0.476					2.458		−35.394	6.217
***Yule3rate***	25.806	0.363	19.017	0.404				1.687	1.680	−41.611	0

Clade B											
Model	LH	r1	r2	r3	a	xp	k	st1	st2	AIC	ΔAIC_rc_
***Pb***	1.844	0.484								−1.688	0
***Bd***	1.844	0.484			0					0.312	2
***DDX***	1.914	0.682				0.179				0.171	−1.859
***DDL***	2.017	0.678					28.304			−0.034	−1.654
***Yule2rate***	2.117	0.640	0.412					1.770		1.766	−3.454
***Yule3rate***	4.663	0.515	19.419	0.412				1.779	1.770	0.673	−2.361

Notes: Analyses were conducted using the ultrametric tree obtained in BEAST. All outgroups and intermittent taxa were pruned to obtain the ingroup clades A and B. Abbreviations: Pb – pure birth; Bd – birth-death; DDX – exponential density dependent; DDL – logistic density dependent; Yule2rate – Yule with 2 rates; Yule3rate – Yule with 3 rates; LH – log-likelihood; r1/r2/r3 – net diversification rates; a – extinction fraction; xp – rate change parameter DDX model; k – carrying capacity parameter DDL model; st1/st2 – timing of rate shift (mya); AIC – Akaike information criterion; ΔAICrc  =  difference between the best rate constant and rate variable models.

### Ancestral Range Reconstruction

Results of ancestral area reconstruction using the dispersal-extinction-cladogenesis model (*dec*-model, LAGRANGE) and statistical Dispersal-Vicariance analysis (RASP) were highly congruent, with four recovered differences in optimal resolutions (bold nodes in Figure 2 and Table S5). Results for reconstruction using the *dec*-model are presented in Figure 2, with asterisks indicating nodes for which multiple equally optimal resolutions were retrieved in Lagrange and/or RASP. Ancestral ranges in Continental Africa and Madagascar were reconstructed for the crown node of Clade A (node 4), following a previous dispersal event from Continental Africa to Madagascar (between nodes 3 and 4). At least four other such cases were documented for clades with reconstructed ancestral areas in Continental Africa dispersing directly to Madagascar (between nodes 39* and 40*, 37 and 38, 43 and 44, and following the split between *Conyza aegyptiaca* and *C. ulmifolia*). A dispersal from Continental Africa to the Comoros is inferred between node 4 and 5. One descendent lineage of node 4 has speciated extensively into clusters of closely related and geographically distinct species in Madagascar (starting with node 7). Over the course of clade A evolution on Madagascar, descendent lineages dispersed to Continental Asia (between nodes 11 to 13 and beyond), to Rodrigues (between nodes 9 and 15) and Europa island and Continental Africa (following the split at node 32) from Madagascar, to Mauritius from Rodrigues (nodes 15 to 16) and finally to Réunion from Mauritius (between nodes 22 and 24). A dispersal from Madagascar underlies the origin of clade B on Réunion (node 45), with Continental Africa reconstructed as the most probable ancestral area for nodes 42 and 43 (Figure 2).

## Discussion

### Biogeography and diversification in the MIOI

Our study has produced the first extensive molecular phylogeny for Indian Ocean daisy trees and related genera, providing strong support for its non-monophyly with a separation into two major clades and several much smaller groups. Results support the hypothesis of a continental African origin for *Psiadia* s. l. and an origin for the majority of species within the last ∼15 Ma, with clades consisting of Mascarene endemic species being the youngest (3.5 & 5.1 Ma). Our results highlight that the Mascarenes were colonized by two evolutionary very distinct lineages from Madagascar, in two independent dispersal events, that are unique for both their spatial and temporal properties. One striking aspect of diversification within our study group is the general absence of interisland dispersal and speciation. This is similar to other key studies on island endemic Asteraceae, such as the Hawaiian Silverswords [115], but differs from examples from the Macaronesian islands (Woody *Sonchus* and other genera [116]; *Argyranthemum* [117]) where interisland dispersal and speciation appear to be the rule rather than the exception. This is not restricted to just Asteraceae however, and is also apparent in other families (for example Monimiaceae, Myrsinaceae, Malvaceae, Rutaceae and many others) suggesting that factors pertaining to the mode and tempo of archipelago formation and its geographical isolation, strongly govern evolutionary pathways available for species diversification.

Apart from the case of Réunion where the ancestors of clade A and B arrived independently, and the accession of South African *P. punctulata*, we found no evidence of either multiple independent dispersals from a source area (such as Madagascar), or back dispersals to the source area by a descendant lineage. Insular lineages of Asteraceae provide many examples of repeated and often high rates of diversification following colonization (see [118] for an extensive summary). Observed high diversification rates for *Psiadia* in clades A and B and the repeated pattern of regional subclade diversification are suggestive of a general propensity for cladogenesis when exposed to isolated habitat and when confronted with initial low population densities. A large number of extensively radiating island endemic Asteraceae have been described in previous studies [119]–[123] and like in our study group, the in situ evolution towards woodiness in island and upland mountain lineages, from herbaceous continental ancestors, is a repeated pattern in Asteraceae and other families [124].

### Dispersal from Africa to Madagascar and diversification in Madagascar

Age estimates for the most recent common ancestor of *Psiadia* species in clade A shows that Madagascar was colonized as recently as the Late Miocene 11.11–10.08 Ma (nodes 3–4; Figures 2 and 3 and Table 1), which is well after its separation from both the African mainland (165–121 Ma) and India (88–63 Ma) [125], [126]. Furthermore, the basal position of Comoron lineages like *P. pascalii* and the geographic distribution of associated species on the Comoros and in northern Madagascar point to an African origin, either direct or via dispersal across the Mozambique Strait islands. Results from ancestral area reconstructions confirm the African origin of both A and B clades, although the role of the Comoros as a dispersal-stepping-stone remains unconfirmed (Table S3) and will warrant further study by inclusion of *P. volubilis*, mainland African species and other species from northern Madagascar. The reconstructed dispersal to the Comoros (between nodes 4 and 5) is supported by studies postulating the existence of land area in the Mozambique Strait for at least the past 10(−15) My [127], although Mayotte itself is thought to be around 7.7 My old ([128] but see [129]).

Dispersal to Madagascar led to a period of relative stasis (period between 10.08 and 4.58 (−3.51) Ma) in which only the ancestral lineages of main species clusters were formed. It was not until 4.58–3.51 Ma that these lineages began to generate the species clusters we recognize today (nodes 33, 10 and 27; Figures 2 and 3; rate shifts B and RC2). The timing of these rate shifts and the coincident speciation bursts in Madagascan *Psiadia* coincide with similar events in other highly diverse plant lineages occurring in Madagascar (e.g. scaly tree ferns [26]). As a result of climatic oscillations during the Pliocene [130] resulting in habitat fragmentation, and subsequent repeated contraction and expansion of restricted forest refugia, it is postulated that these taxa underwent rapid, geographically extensive and concomitant diversification bursts. We hypothesize that a similar scenario may have induced rapid cladogenesis in ancestral Madagascan *Psiadia*, with a crucial role for the effects of shifting altitudinal gradients on the creation of isolated and vicariant populations in Madagascar's northern and eastern mountain ranges. Such an evolutionary scenario would create a pattern of repeated regional diversification, characterized by morphologically highly similar but evolutionary and biogeographically distinct subclades. For example, the most basal cluster of clade A (Figure 2 and 3) consists of species that only occur in the far north of Madagascar at very high elevations in the lower sclerophyllous montane forest and ericoid montane thickets of the Marojejy massif [110].

To test whether this scenario applies to our study group and other taxa on Indian Ocean islands, and to fully understand the effects such shifting altitudinal gradients may have had on the distribution, isolation and diversification of upland ancestral *Psiadia*, we would need to include the remaining (far-) northern and eastern species of Madagascar in follow-up analyses and reconstructions. If our hypothesis is correct, we would expect to find additional clusters of biogeographically distinct, upland species originating in the Pliocene (but with a single ancestral lineage originating considerably before then as evidenced by long branches subtending nodes 10, 27 and 33 – Figure 2). Furthermore, a follow-up study using population genetics and focusing on species placed in the Marojejy-group may shed more light on the occurrence, frequency and length of such movements and isolations, and the speed and genetic extent of lineage divergence within this mountain range.

### Dispersal from Madagascar to the Mascarenes and diversification in the Mascarenes

A second round of diversification in clade A took place in the early Mascarenes following dispersals to Rodrigues (between 5.38–3.51 Ma), Mauritius (3.51–2.33 Ma) and finally to Réunion (1.35–1.00 Ma). Our results provide evidence that shifts in diversification rates occurred in clade A in response to geographic range expansion and, although we were unable to include all Madagascan species in this study, we hypothesize that a significant rate shift may have occurred early in the radiation of clade A, as suggested when we took under sampling into account using the γ-estimate [103].

The ancestor of the second lineage to colonize the Mascarenes (clade B) dispersed to Réunion from Madagascar in the Early Pliocene, and radiated into a wide range of primarily mid- and high-elevation habitats. Short internodal branch lengths to its nearest relative (*Conyza neocandolleana* from Madagascar) indicate that diversification started soon after colonization, but the majority of divergence events are relatively young (10/14 <1 Ma). The causes for this pattern may be found in the early phase of island formation.

The physical geography of volcanic islands in their early phases, such as Réunion at the time of colonization, is both highly complex and dynamic as set forth by Whittaker *et al*. [28]. The authors formulate a general dynamic model (GDM) of oceanic island biogeography, incorporating the relationships between three key biogeographical processes (speciation, immigration, extinction) over geological time and in relation to island ontogeny to explain patterns of biodiversity which we found to apply closely for the Mascarenes and in particular Réunion. The model distinguishes four temporal phases of island evolution (*youth*; *immaturity*; *maturity*; *old age*) and goes into detail of how rates (speciation (*s*), immigration (*i*) and extinction (*e*)) and species numbers (potential carrying capacity (*K*) and realized species richness (R)) change over the course of an island's geological life cycle (see Figure 4 in [28]). Pertinent to the case of Réunion, the model predicts that the maximum area and elevational range of an island are reached just before maximum topographic complexity is obtained, at which point the maximum potential carrying capacity is realized (K). Higher islands can support more diverse ranges of habitat than lower altitude islands facilitating the existence of more endemic species [131], however, the potential carrying capacity of an island can suffer from large setbacks in the *youth* and *old age* phases due to violent volcanic activity and large destructive landslides, as appears to have happened repeatedly on Réunion [132]–[134].

In the maturity phase the total number of species present on an island reaches its maximum attainable number (*R*). As the island begins to show the first major signs of erosion shortly after active volcanic buildup and renewal ceases, speciation rates remain high as the formation of increasingly deep valleys offer opportunities for micro-allopatric speciation of fragmented populations on a fine geographic scale. The current topography of Réunion offers a good example of this phase with over two-thirds of the island covered by the three large calderas of the Piton des Neiges (∼3000 m) which underwent a series of three explosive episodes of volcanic activity some 230,000–180,000 years ago [135] with the most recent major eruptions occurring some 12,000 years ago [132]. The island's interior is now dissected by impressively steep cliffs and deep ravines, the latter serving as drainage pathways taking out large quantities of sediment and erosion material.

Random extinction of early upland lineages (by periods of extensive volcanic activity) and renewed cladogenesis in later stages (via micro-allopatric speciation of fragmented populations) would obscure part of the original phylogenetic pattern of diversification and carry the signal of a more recent wave of diversification within island clades, characterized by young species and low sequence divergence. On young volcanic islands such as Réunion, levels of habitat diversity are high with many closely related novel forms present. On older volcanic islands, such as Mauritius, habitat diversity has been in decline for a long time and current plant communities are remnants of a once rich and varied system of habitat types stretching from sea level to high altitudes (like the high-altitude ericaceous vegetation still present on Réunion (∼2500–3000 m). Relictual populations of endemic upland species on Mauritius at the last remaining high elevation habitat (<828 m) are witness to this and have become ecologically marginalized over the course of the evolutionary and geological life cycle of an oceanic island. Similar patterns of in situ diversification and the coexistence of closely related, geographically proximate yet biological distinct species have been found on the Mascarenes in other taxa (Myrsinaceae [72]; Ebenaceae – J.S. Strijk, unpublished data; Monimiaceae [73]; Malvaceae [136] and others). The majority of these endemic species have naturally low population densities making them particularly vulnerable in light of the myriad of problems facing oceanic island biota (e.g. habitat conversion, over collecting, invasive species). Little to no data is available on the remaining numbers for most of these species [72], [137], and much of that knowledge is retained by a handful of botanists who reside on the islands in question.

### Dispersal tracks through the Indian Ocean basin and the role of Rodrigues

In clade A, the inferred sequence and age estimates for colonization and diversification lend support to a more ancient origin of Rodrigues than was previously thought by some to be the case [68], [70]. Such an ancient origin is in line with certain historical and geophysical records, plus biological observations and recent hypotheses [138]–[141]. Moreover, similar biogeographical scenarios to that outlined here for *Psiadia* have been observed in other regionally species rich groups (e.g., Myrsinaceae, [72] and J.S. Strijk. unpublished data; Monimiaceae, [73]).

Some uncertainty regarding the exact age of Rodrigues and more importantly its role in regional biotic assembly remains, as little direct evidence in the form of geological data are available. The islands do not share a similar geological origin [70], with Rodrigues located at a tri-plate fracture whereas Mauritius (and Réunion) are the result of plate movement over the Réunion volcanic hotspot. This difference in origin may have had a profound impact on the timing, manner and speed with which the respective islands appeared, the ecological opportunities available for colonizing taxa during the initial phase, and the extent and impact of cataclysmic volcanic eruptions on early biota, as has been shown to have occurred on both Mauritius and Réunion [69], [133], [142]. The latter may have resulted in high levels of extinction across fauna and flora assemblages on each of the islands, although studies have shown that not all lineages are erased by such events [143], [144]. An additional complicating factor lies in the highly degraded state of Rodrigues (both geological and man-induced) which has resulted in high levels of extinction and hampers efforts to reconstruct the formation of its biota [70]. Despite these problems most authors now believe that the age of Rodrigues must be at least that of Mauritius (between 8–15 My) [23].

The occurrence of extensive sea-level fluctuations and the documented existence of large numbers of islands along the Nazareth and Saya de Malha banks (as early as the late Oligocene and as recently as the Pleistocene (2.4–1.6 Ma) are expected to have played a major role in primary biotic assembly and ultimately the opportunities for diversification on these islands [17], [130], [145]–[147]. Colonist lineages of certain groups may have persisted on presently submerged islands before modern day island chains arose from the sea (e.g. geckos [143]; palms [148]; Monimiaceae [73]). In addition, other species rich groups have been identified that are likely to have used these low lying island areas to disperse into the Western Indian Ocean from other islands and continental landmasses in the northeast and east (e.g. Dodo (*Raphus*) and relatives [147]; Bulbuls (*Hypsipetes*) [14]; bats (*Emballonura*) [149] and magpie-robins (*Copsychus*) [150]).

### Causes for species accumulation on peripheral Indian Ocean islands

The unidirectional pattern of dispersal and radiation of *Psiadia* in Clade A (i.e. from Madagascar to Rodrigues to Mauritius to Réunion) and the single dispersal event leading to the radiation of clade B (Madagascar > Réunion) have both given rise to fourteen endemic species. Although similar in absolute numbers, members of clade B on Réunion are unique in the sense that all species of this clade inhabit the same island (and are endemic to it), and occur in unique zonal clusters along an altitudinal cline. Together, diversification events in the Mascarene Islands account for at least 28 species of *Psiadia* (via four unique dispersals), which is likely to be a conservative estimate given natural species decline with oceanic island age [28], and evidence for widespread extinction of species on Mauritius and Rodrigues due to human activities in the last three centuries [70]. *Psiadia* is estimated to have been present on Madagascar from 11.11–10.08 Ma (dispersal between node 3 and 4, Figure 2), and the number of taxa confined to Madagascar account for *ca.* 30 species (including taxa not sampled in this study). On peripheral islands, *Psiadia* has been present from 7.35–5.11 Ma (Clade A – Madagascar > Réunion) or 3.51–5.38 Ma (Clade B – Madagascar > Rodrigues). With Madagascar being over 100 times the size of the combined surface area of the Mascarene islands, and with habitat conditions on volcanic Indian Ocean islands of a considerably more transient nature (both on a geological, temporal and environmental scale) [28], current patterns of species diversity suggest that lineages experienced rather different dynamics of speciation and extinction on Madagascar and its peripheral islands. One way to better understand these different dynamics is by looking at examples of diversification in isolation on islands at the other extreme of island size ranges found in the Western Indian Ocean.

We included in our study an accession of *Psiadia altissima*, which occurs on both Madagascar and Europa island in the Mozambique Straits. The latter island is interesting for our understanding of dispersal and diversification in the region in the sense that despite its small size (28 km^2^) and simple topography, it too holds a number of endemic subspecies and races (mostly of birds, reptiles and invertebrates). Comparable patterns of endemism can be found on many other small, but highly isolated islands in the Western Indian ocean (e.g. Juan de Nova island, Glorioso islands, Assumption, Tromelin and Agalega) [151]. Although poorly studied, hitherto cases of young island endemism (at low taxonomic levels, yet genetically distinct) provide an insight into how *waif* dispersal operates frequently enough to reach and populate highly isolated islands, yet be sufficiently rare to allow for drift and diversification [152]. Such examples suggest that even on islands that are ecologically simple and very limited in size, the combined effects of strong isolation and biological filtering, time and a simplified pallet of ecological pressures provide the starting conditions for rapid evolutionary modification of new arrivals.

## Supporting Information

Figure S1 **Bayesian 50% majority rule consensus tree with associated branch lengths.** Notes: Branch labels indicate maximum likelihood bootstrap values followed by Bayesian posterior probabilities (in bold) from MrBayes. Circles indicate support for resolved nodes, as opposed to a polytomy, estimated by PHYCAS (black: 0.91–100; blue: 0.76–0.90; open: 0.60–0.75).
(TIF)

Table S1 **Specimens, voucher information and GenBank accessions.** Notes: Classifications of the Flore des Mascareignes [109] and Madagascar Catalogue [112] are followed. Leaf samples and collections obtained from field (Toulouse DNA-bank (TDNA)), the DNA Bank at Royal Botanic Gardens, Kew (KDB), SANBI at Kirstenbosch (LHMS), or from GenBank (GB). For taxa obtained from botanical gardens, localities indicate source of introduction. Accessions included in final analyses are highlighted with *.
(DOC)

Table S2 **Primer pairs used in this study.**
(DOC)

Table S3 **DNA isolation, purification and PCR conditions.** Notes: Total DNA was isolated using the BioSprint DNA Plant Kit (Qiagen, Valencia, CA) with a modified protocol, using ball bearings and silica powder. All markers were amplified from genomic DNA using PCR. Each 25 µl reaction contained 0.1 µl (5.0 units) of *GoTaq* polymerase (Promega, Madison, WI), 5 µl of 5x reaction buffer, 0.5 µl dNTP (10 mM stock solution), 0.5 µl of each primer (10mM stock solution) and 1.0 µl template DNA. PCR products were cleaned using the MinElute PCR Purification Kit (Qiagen, Valencia, CA). Sequencing was performed on an ABI 3730xl (Genoscope, Evry, France).
(DOC)

Table S4 **Calibration scheme and results of molecular dating analyses for selected nodes using Penalized Likelihood and BEAST.** Notes: Values in millions of years ago. Abbreviations: HPD – Highest Posterior Density interval; (logN) – logNormal prior; (N) – Normal prior; PL – Penalized Likelihood; PP – Posterior Probability; UCLN – uncorrelated lognormal; (U) – Uniform prior.
(DOC)

Table S5 **Inferred ancestral ranges for branches** (**separated by vertical bar**) **descending from each node in** **Figure 2** **, and their relative probability.** Note: Only alternative scenarios that fall within two log-likelihood units of the optimal reconstruction and have a relative probability ≥0.1 are provided. Asterisks highlight nodes for which multiple equally probable solutions were retrieved using Lagrange and/or S-DIVA. Clades for which differences exist in optimal reconstructions between Lagrange and S-DIVA are shown in bold.
(DOC)
