# Correction: In and out of Madagascar: Dispersal to Peripheral Islands, Insular Speciation and Diversification of Indian Ocean Daisy Trees (*Psiadia*, Asteraceae)

**DOI:** 10.1371/annotation/fa4d2334-e48c-4e29-b8e1-07d755d317fa

**Published:** 2013-08-01

**Authors:** Joeri S. Strijk, Richard D. Noyes, Dominique Strasberg, Corinne Cruaud, Fredéric Gavory, Mark W. Chase, Richard J. Abbott, Christophe Thébaud

Table S1's accession list is currently missing the accession numbers. Please see the correct version of Table S1 with the accession numbers at the following link:

http://www.plosone.org/corrections/pone.0042932.cn.s001.doc 

