# Supplementary figures and images for: In and out of Madagascar: Dispersal to Peripheral Islands, Insular Speciation and Diversification of Indian Ocean Daisy Trees (Psiadia, Asteraceae)
Source: PLoS One. 2012 Aug 10;7(8):e42932. doi: 10.1371/journal.pone.0042932 (PMC3416790; doi:10.1371/journal.pone.0042932)

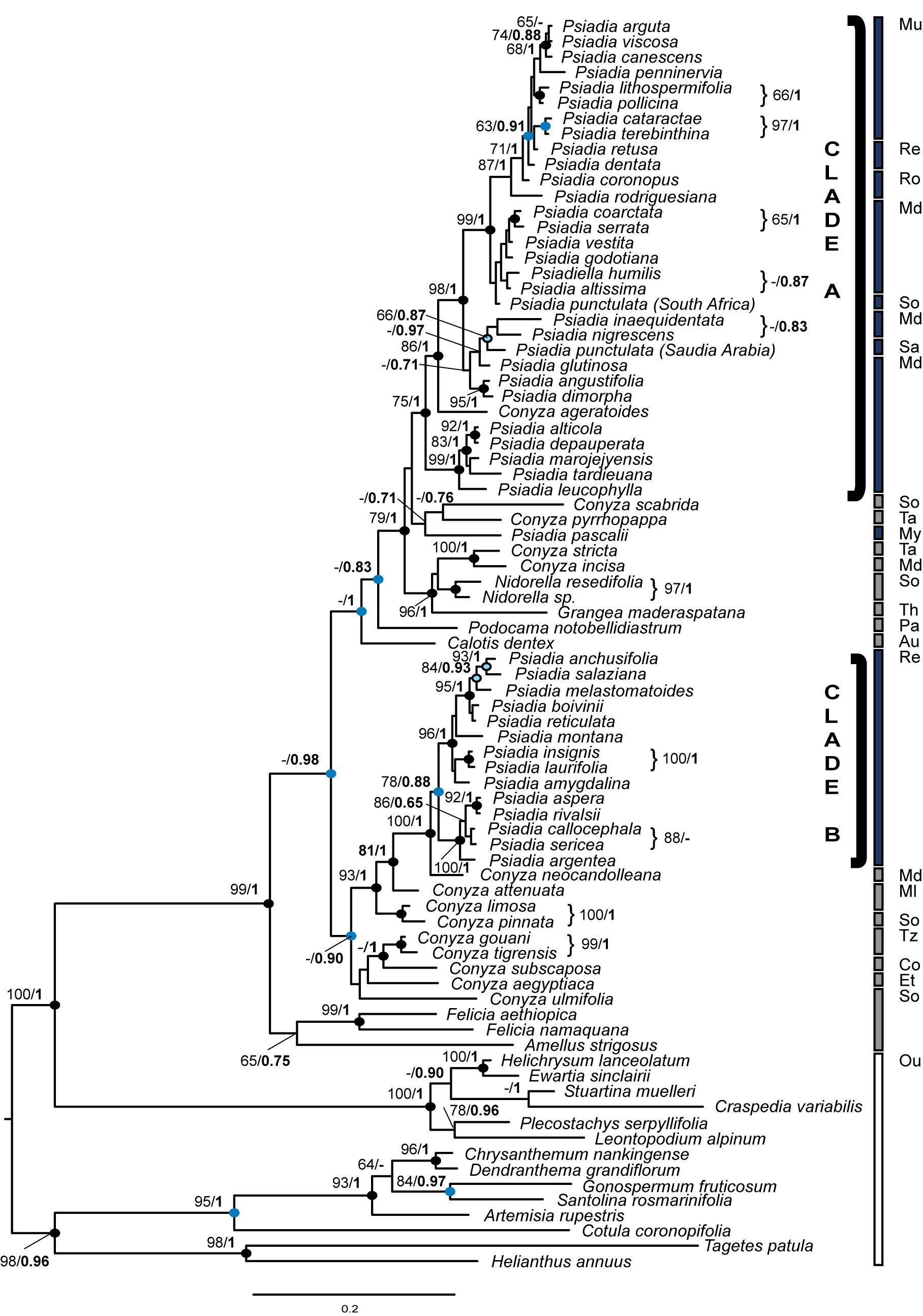

Supplement: Figure S1 — Bayesian 50% majority rule consensus tree with associated branch lengths. Notes: Branch labels indicate maximum likelihood bootstrap values followed by Bayesian posterior probabilities (in bold) from MrBayes. Circles indicate support for resolved nodes, as opposed to a polytomy, estimated by PHYCAS (black: 0.91–100; blue: 0.76–0.90; open: 0.60–0.75). (TIF) [file pone.0042932.s001.tif]
