# Supplementary material for: In and out of Madagascar: Dispersal to Peripheral Islands, Insular Speciation and Diversification of Indian Ocean Daisy Trees (Psiadia, Asteraceae)
Source: PLoS One. 2012 Aug 10;7(8):e42932. doi: 10.1371/journal.pone.0042932 (PMC3416790; doi:10.1371/journal.pone.0042932)
Supplement: Table S1 — Specimens, voucher information and GenBank accessions. Notes: Classifications of the Flore des Mascareignes [109] and Madagascar Catalogue [112] are followed. Leaf samples and collections obtained from field (Toulouse DNA-bank (TDNA)), the DNA Bank at Royal Botanic Gardens, Kew (KDB), SANBI at Kirstenbosch (LHMS), or from GenBank (GB). For taxa obtained from botanical gardens, localities indicate source of introduction. Accessions included in final analyses are highlighted with *. (DOC) [file pone.0042932.s002.doc]

| **Taxon** | **Accession number** | **Collection locality data** | **GenBank** |  |  |  |
| --- | --- | --- | --- | --- | --- | --- |
|  |  |  | *acc*D | *rpo*B | *psb*A-*trn*H | ITS |
| *Psiadia agatheoides* (Cass.) Drake | TDNA631 | Pic St-Louis, Tôlanaro, Madagascar | EU###### | EU###### | EU###### | EU###### |
| *Psiadia agatheoides* (Cass.) Drake | TDNA632 | Pic St-Louis, Tôlanaro, Madagascar | EU###### | - | EU###### | - |
| *Psiadia alticola* Humbert | TDNA636 | Marojejy, Madagascar | EU###### | - | - | EU###### |
| *Psiadia alticola* Humbert | TDNA637* | Marojejy, Madagascar | EU###### | EU###### | EU###### | EU###### |
| *Psiadia alticola* Humbert | TDNA639 | Marojejy, Madagascar | EU###### | EU###### | EU###### | EU###### |
| *Psiadia altissima* (DC.) Drake | TDNA634 | Talakifeno, Madagascar | EU###### | - | EU###### | EU###### |
| *Psiadia altissima* (DC.) Drake | TDNA635 | Talakifeno, Madagascar | EU###### | - | EU###### | EU###### |
| *Psiadia altissima* (DC.) Drake | TDNA666 | Bevilany, Madagascar | EU###### | - | EU###### | EU###### |
| *Psiadia altissima* (DC.) Drake | TDNA667 | Bevilany, Madagascar | - | - | EU###### | EU###### |
| *Psiadia altissima* (DC.) Drake | TDNA668 | Bevilany, Madagascar | - | - | EU###### | EU###### |
| *Psiadia altissima* (DC.) Drake | TDNA669 | Ankororoka, Madagascar | - | - | EU###### | EU###### |
| *Psiadia altissima* (DC.) Drake | TDNA670* | Ankororoka, Madagascar | EU###### | EU###### | EU###### | EU###### |
| *Psiadia altissima* (DC.) Drake | TDNA763 | Europa Island | - | - | EU###### | EU###### |
| *Psiadia amygdalina* Cordem. | TDNA764 | Ravine Savane Cimetière, Ste-Rose, Réunion | EU###### | EU###### | EU###### | - |
| *Psiadia amygdalina* Cordem. | TDNA765* | Cap Noir, La Possession, Réunion | EU###### | EU###### | EU###### | EU###### |
| *Psiadia anchusifolia* Cordem. | TDNA767* | Nez de Boeuf, Le Tampon, Réunion | - | - | EU###### | EU###### |
| *Psiadia anchusifolia* Cordem. | TDNA768 | Bébour, St-Benoit, Réunion | - | - | EU###### | EU###### |
| *Psiadia anchusifolia* Cordem. | TDNA770 | La Roche Ecrite, St-Denis, Réunion | - | - | EU###### | EU###### |
| *Psiadia anchusifolia* Cordem. | TDNA773 | La Petite France, St-Paul, Réunion | - | - | EU###### | EU###### |
| *Psiadia anchusifolia* Cordem. | TDNA775 | Le Petit Matarum, Cilaos, Réunion | - | - | EU###### | EU###### |
| *Psiadia angustifolia* (Humbert) Humbert | TDNA640* | Baie des Galions, Tôlanaro, Madagascar | EU###### | EU###### | EU###### | EU###### |
| *Psiadia angustifolia* (Humbert) Humbert | TDNA641 | Baie des Galions, Tôlanaro, Madagascar | EU###### | EU###### | EU###### | EU###### |
| *Psiadia angustifolia* (Humbert) Humbert | TDNA642 | Baie des Galions, Tôlanaro, Madagascar | EU###### | EU###### | EU###### | EU###### |
| *Psiadia argentea* Cordem. | TDNA777 | La Roche Ecrite, St-Denis, Réunion | EU###### | EU###### | EU###### | EU###### |
| *Psiadia argentea* Cordem. | TDNA778 | Bois Ozoux, Le Tampon, Réunion | EU###### | EU###### | EU###### | EU###### |
| *Psiadia argentea* Cordem. | TDNA779 | Le Grand Bord, St-Paul, Réunion | EU###### | EU###### | EU###### | EU###### |
| *Psiadia argentea* Cordem. | TDNA780 | Nez de Boeuf, Le Tampon, Réunion | EU###### | EU###### | EU###### | EU###### |
| *Psiadia argentea* Cordem. | TDNA781 | Cavernes du Bras Chanson, St-Benoit, Réunion | EU###### | EU###### | EU###### | EU###### |
| *Psiadia argentea* Cordem. | TDNA782* | Caverne Dufour, Cilaos, Réunion | EU###### | EU###### | EU###### | EU###### |
| *Psiadia arguta* Voigt | KDB25778* | Ile Plate, Outer Islets, Mauritius (cultivated, Mondrain Nature Reserve) | EU###### | EU###### | EU###### | EU###### |
| *Psiadia arguta* Voigt | TDNA783 | Ile Plate, , Outer Islets, Mauritius (cultivated, University of Réunion) | - | - | EU###### | - |
| *Psiadia arguta* Voigt | TDNA784 | Ile Plate, , Outer Islets, Mauritius (cultivated, University of Réunion) | - | - | EU###### | EU###### |
| *Psiadia aspera* Cordem. | TDNA787* | Savane Cimetière, Ste-Rose, Réunion | EU###### | EU###### | EU###### | EU###### |
| *Psiadia aspera* Cordem. | TDNA788 | Savane Cimetière, Ste-Rose, Réunion | EU###### | EU###### | - | EU###### |
| *Psiadia boivinii* B.L.Rob. | TDNA789 | Basse Vallée, St-Philipe, Réunion | - | - | EU###### | EU###### |
| *Psiadia boivinii* B.L.Rob. | TDNA790* | Vallée Heureuse, St-Philippe, Réunion | - | - | EU###### | EU###### |
| *Psiadia callocephala* Cordem. | TDNA791 | Piton dans l’Bout, Le Tampon, Réunion | - | - | EU###### | EU###### |
| *Psiadia callocephala* Cordem. | TDNA792 | Le Grand Bénare, Les Trois-Bassins, Réunion | EU###### | EU###### | EU###### | EU###### |
| *Psiadia callocephala* Cordem. | TDNA793 | Fomica Leo, Ste-Rose, Réunion | EU###### | EU###### | EU###### | EU###### |
| *Psiadia callocephala* Cordem. | TDNA794 | Cratère Commerson, St-Joseph, Réunion | EU###### | EU###### | EU###### | EU###### |
| *Psiadia callocephala* Cordem. | TDNA795 | Caverne Dufour, Cilaos, Réunion | EU###### | EU###### | EU###### | EU###### |
| *Psiadia callocephala* Cordem. | TDNA796 | Piton des Neiges, Cilaos, Réunion | EU###### | EU###### | EU###### | EU###### |
| *Psiadia callocephala* Cordem. | TDNA797* | Le Grand Bénare, Les Trois-Bassins, Réunion | EU###### | EU###### | EU###### | EU###### |
| *Psiadia canescens* A.J.Scott | TDNA798* | Piton du Fouge, Black River, Mauritius | EU###### | EU###### | EU###### | EU###### |
| *Psiadia cataractae* A.J.Scott | TDNA799 | Tamarind Falls, Plaines Wilhems, Mauritius | EU###### | EU###### | EU###### | EU###### |
| *Psiadia cataractae* A.J.Scott | TDNA800* | Tamarind Falls, Plaines Wilhems, Mauritius | EU###### | EU###### | EU###### | EU###### |
| *Psiadia coarctata* (Humbert) Humbert | TDNA643* | Lake Anony, Madagascar | EU###### | EU###### | EU###### | EU###### |
| *Psiadia coarctata* (Humbert) Humbert | TDNA644 | Lake Anony, Madagascar | EU###### | EU###### | EU###### | EU###### |
| *Psiadia coronopus* Hook.f. & Benth. ex. Balf.f. | TDNA832* | Rodrigues (cultivated Mascarin Botanic Garden, Réunion Island) | EU###### | EU###### | EU###### | EU###### |
| *Psiadia dentata* DC. | TDNA801 | Cap Noir, La Possession, Réunion | EU###### | EU###### | EU###### | EU###### |
| *Psiadia dentata* DC. | TDNA802* | Cap Noir, La Possession, Réunion | EU###### | EU###### | EU###### | EU###### |
| *Psiadia depauperata* Humbert | TDNA645* | Marojejy, Madagascar | EU###### | EU###### | EU###### | EU###### |
| *Psiadia depauperata* Humbert | TDNA646 | Marojejy, Madagascar | EU###### | EU###### | EU###### | EU###### |
| *Psiadia depauperata* Humbert | TDNA803 | Marojejy, Madagascar | EU###### | EU###### | EU###### | EU###### |
| *Psiadia depauperata* Humbert | TDNA804 | Marojejy, Madagascar | EU###### | EU###### | EU###### | EU###### |
| *Psiadia dimorpha* Humbert | TDNA647* | Cap Sainte Marie, Madagascar | EU###### | EU###### | EU###### | EU###### |
| *Psiadia dimorpha* Humbert | TDNA648 | Cap Sainte Marie, Madagascar | EU###### | EU###### | EU###### | EU###### |
| *Psiadia dimorpha* Humbert | TDNA649 | Cap Sainte Marie, Madagascar | EU###### | EU###### | EU###### | EU###### |
| *Psiadia glutinosa* Jacq. | TDNA650* | Tôlanaro, Madagascar | EU###### | EU###### | EU###### | EU###### |
| *Psiadia glutinosa* Jacq. | TDNA651 | Tôlanaro, Madagascar | EU###### | EU###### | EU###### | EU###### |
| *Psiadia glutinosa* Jacq. | TDNA652 | Tôlanaro, Madagascar | EU###### | EU###### | EU###### | EU###### |
| *Psiadia godotiana* Humbert | TDNA653* | Mont Vatazo, Talakifeno, Madagascar | - | - | EU###### | EU###### |
| *Psiadia godotiana* Humbert | TDNA654 | Mont Vatazo, Talakifeno, Madagascar | - | - | EU###### | - |
| *Psiadia inaequidentata* Humbert | TDNA655 | Pic St-Louis, Tôlanaro, Madagascar | EU###### | EU###### | EU###### | EU###### |
| *Psiadia inaequidentata* Humbert | TDNA656 | Pic St-Louis, Tôlanaro, Madagascar | EU###### | EU###### | EU###### | EU###### |
| *Psiadia inaequidentata* Humbert | TDNA657* | Pic St-Louis, Tôlanaro, Madagascar | EU###### | EU###### | EU###### | EU###### |
| *Psiadia insignis* Cordem. | TDNA805* | Ravine Savane Cimetière, Ste-Rose, Réunion | - | - | EU###### | EU###### |
| *Psiadia insignis* Cordem. | TDNA806 | Ravine Savane Cimetière, Ste-Rose, Réunion | EU###### | EU###### | EU###### | - |
| *Psiadia laurifolia* Cordem. | TDNA807 | Basse Vallée, St-Philipe, Réunion | EU###### | EU###### | EU###### | EU###### |
| *Psiadia laurifolia* Cordem. | TDNA808* | Basse Vallée, St-Philipe, Réunion | EU###### | EU###### | EU###### | EU###### |
| *Psiadia leucophylla* (Baker) Humbert | TDNA662 | Marojejy, Madagascar | EU###### | EU###### | EU###### | EU###### |
| *Psiadia leucophylla* (Baker) Humbert | TDNA663 | Marojejy, Madagascar | EU###### | EU###### | EU###### | EU###### |
| *Psiadia leucophylla* (Baker) Humbert | TDNA665* | Marojejy, Madagascar | EU###### | EU###### | EU###### | EU###### |
| *Psiadia lithospermifolia* Cordem. | TDNA810 | Morne Brabant, Black River, Mauritius | - | - | EU###### | EU###### |
| *Psiadia lithospermifolia* Cordem. | TDNA811* | Morne Brabant, Black River, Mauritius | EU###### | EU###### | EU###### | EU###### |
| *Psiadia marojejyensis* (Humbert) Humbert | TDNA671 | Marojejy, Madagascar | EU###### | EU###### | EU###### | EU###### |
| *Psiadia marojejyensis* (Humbert) Humbert | TDNA673 | Marojejy, Madagascar | EU###### | EU###### | EU###### | EU###### |
| *Psiadia marojejyensis* (Humbert) Humbert | TDNA674* | Marojejy, Madagascar | EU###### | EU###### | EU###### | EU###### |
| *Psiadia melastomatoides* (Lam.) A.J.Scott | TDNA812* | Le Petit Matarum, Cilaos, Réunion | EU###### | EU###### | EU###### | EU###### |
| *Psiadia melastomatoides* (Lam.) A.J.Scott | TDNA814 | Pas de Bellecombe, Ste-Rose, Réunion | EU###### | EU###### | EU###### | EU###### |
| *Psiadia melastomatoides* (Lam.) A.J.Scott | TDNA815 | Le Coteau Maigre, St-Benoit, Réunion | EU###### | EU###### | - | EU###### |
| *Psiadia montana* Baill. | TDNA817* | Cap Noir, La Possession, Réunion | - | - | EU###### | EU###### |
| *Psiadia nigrescens* Humbert | TDNA679 | Montagne des Français, Antsiranana, Madagascar | EU###### | EU###### | EU###### | EU###### |
| *Psiadia nigrescens* Humbert | TDNA680* | Montagne des Français, Antsiranana, Madagascar | EU###### | EU###### | EU###### | EU###### |
| *Psiadia nigrescens* Humbert | TDNA682 | Montagne des Français, Antsiranana, Madagascar | EU###### | EU###### | EU###### | EU###### |
| *Psiadia pascalii* Labat & Beentje | TDNA813* | Mlima Choungui, Mayotte | - | - | EU###### | EU###### |
| *Psiadia penninervia* DC. | KDB25898 | Perrier Reserve, Plaines Wilhems, Mauritius (cultivated in RBGKew, Living Coll.1995-3377 ex Conservatoire Botanique National de Mascarin, Acc.No.921672) | EU###### | EU###### | EU###### | EU###### |
| *Psiadia penninervia* DC. | KDB27719 | RBGKew, Living Coll.1975-39, Réunion | EU###### | EU###### | EU###### | EU###### |
| *Psiadia penninervia* DC. | TDNA818 | Perrier Reserve, Plaines Wilhems, Mauritius | - | - | EU###### | - |
| *Psiadia penninervia* DC. | TDNA819* | Perrier Reserve, Plaines Wilhems, Mauritius | - | - | EU###### | EU###### |
| *Psiadia pollicina* A.J.Scott | KDB25775 | Le Pouce, Port_Louis, Mauritius (cultivated in Mondrain Nature Reserve, Mauritius) | EU###### | EU###### | EU###### | EU###### |
| *Psiadia pollicina* A.J.Scott | TDNA820* | Le Pouce, Port_Louis, Mauritius | EU###### | EU###### | EU###### | EU###### |
| *Psiadia pollicina* A.J.Scott | TDNA821 | Le Pouce, Port Louis, Mauritius | EU###### | EU###### | EU###### | EU###### |
| *Psiadia punctulata* (DC) Vatke | MO4346138* | South Africa, Transvaal, Zebediela. Brusse 5607 | - | - | - | AF046954 |
| *Psiadia punctulata* (DC) Vatke | TDNA822* | Jabal Banī Sa‘d, Saudi Arabia | EU###### | EU###### | EU###### | EU###### |
| *Psiadia reticulata* A.J.Scott | TDNA809 | Bébour, St-Benoit, Réunion | EU###### | EU###### | EU###### | EU###### |
| *Psiadia reticulata* A.J.Scott | TDNA825* | Bébour, St-Benoit, Réunion | EU###### | EU###### | EU###### | EU###### |
| *Psiadia retusa* DC. | TDNA823* | Bois Blanc, Ste-Rose, Réunion | - | - | EU###### | EU###### |
| *Psiadia retusa* DC. | TDNA824 | Bois Blanc, Ste-Rose, Réunion | - | - | EU###### | EU###### |
| *Psiadia rivalsii* A.J.Scott | TDNA047* | Le Petit Matarum, Cilaos, Réunion | - | - | - | EU###### |
| *Psiadia rodriguesiana* Balf.f. | TDNA830 | Cascade St-Louis, Rodrigues | EU###### | EU###### | EU###### | EU###### |
| *Psiadia rodriguesiana* Balf.f. | TDNA831* | Cascade St-Louis, Rodrigues | - | - | EU###### | EU###### |
| *Psiadia salaziana* Cordem. | TDNA826* | Le Petit Matarum, Cilaos, Réunion | EU###### | EU###### | EU###### | EU###### |
| *Psiadia salaziana* Cordem. | TDNA827 | Le Petit Matarum, Cilaos, Réunion | EU###### | EU###### | EU###### | EU###### |
| *Psiadia salaziana* Cordem. | TDNA829 | Pas de Bellecombe, Ste-Rose, Réunion | EU###### | EU###### | EU###### | EU###### |
| *Psiadia sericea* Cordem. | TDNA835 | Caverne Dufour, Cilaos, Réunion | EU###### | EU###### | EU###### | EU###### |
| *Psiadia sericea* Cordem. | TDNA836 | Le Grand Bénare, Les Trois-Bassins, Réunion | EU###### | EU###### | EU###### | EU###### |
| *Psiadia sericea* Cordem. | TDNA837 | Le Grand Bénare, Les Trois-Bassins, Réunion | EU###### | EU###### | EU###### | EU###### |
| *Psiadia sericea* Cordem. | TDNA839* | Plaine des Sables, Ste-Rose, Réunion | EU###### | EU###### | EU###### | EU###### |
| *Psiadia serrata* (Humbert) Humbert | TDNA683* | Joffreville, Madagascar | EU###### | EU###### | EU###### | EU###### |
| *Psiadia serrata* (Humbert) Humbert | TDNA685 | Joffreville, Madagascar | EU###### | EU###### | EU###### | EU###### |
| *Psiadia serrata* (Humbert) Humbert | TDNA687 | Joffreville, Madagascar | EU###### | EU###### | EU###### | EU###### |
| *Psiadia tardieuana* Humbert | TDNA688 | Marojejy, Madagascar | EU###### | - | - | EU###### |
| *Psiadia tardieuana* Humbert | TDNA690 | Marojejy, Madagascar | EU###### | EU###### | EU###### | EU###### |
| *Psiadia tardieuana* Humbert | TDNA692* | Marojejy, Madagascar | EU###### | EU###### | EU###### | EU###### |
| *Psiadia terebinthina* A.J.Scott | TDNA840 | Le Petrin, Plaines Wilhems, Mauritius | EU###### | EU###### | EU###### | EU###### |
| *Psiadia terebinthina* A.J.Scott | TDNA841* | Le Petrin, Plaines Wilhems, Mauritius | EU###### | EU###### | EU###### | EU###### |
| *Psiadia vestita* Humbert | TDNA693 | Cap Sainte Marie, Madagascar | EU###### | EU###### | EU###### | EU###### |
| *Psiadia vestita* Humbert | TDNA694* | Cap Sainte Marie, Madagascar | EU###### | EU###### | EU###### | EU###### |
| *Psiadia vestita* Humbert | TDNA695 | Cap Sainte Marie, Madagascar | EU###### | - | - | - |
| *Psiadia viscosa* (Lam.) A.J.Scott | TDNA842* | Florin, Plaines Wilhems, Mauritius | EU###### | EU###### | EU###### | EU###### |
| *Psiadia viscosa* (Lam.) A.J.Scott | TDNA843 | Florin, Plaines Wilhems, Mauritius | EU###### | EU###### | EU###### | EU###### |
| *Psiadiella humilis* Humbert | TDNA659 | Cap Sainte Marie, Madagascar | EU###### | EU###### | EU###### | EU###### |
| *Psiadiella humilis* Humbert | TDNA660 | Cap Sainte Marie, Madagascar | EU###### | EU###### | EU###### | EU###### |
| *Psiadiella humilis* Humbert | TDNA661* | Cap Sainte Marie, Madagascar | EU###### | EU###### | EU###### | EU###### |
| *Amellus strigosus* (Thunb.) Less. | MO4354529 | South Africa, Cape, Riversdale. Germishuizen 4204 | - | - | - | AF046942 |
| *Artemisia rupestris* L. | - | - | - | - | - | AJ297261 |
| *Calotis dentex*R.Br. | MO4329780 | Australia, Queensland, Burnett, Toondahra. Forster 5075 | - | - | - | AF046956 |
| *Chrysanthemum nankingense* (Hand.-Mazz.) X.D.Cui | - | - | - | - | - | AF314604 |
| *Conyza aegyptiaca* (L.) Aiton | MO3860153 | Ethiopia, Sidamo, Negele. Friis 3102 | - | - | - | GU045821 |
| *Conyza ageratoides* DC. | MO4576139 | Madagascar, Antsirabe. Du Puy MB332 | - | - | - | GU045822 |
| *Conyza attenuata* DC. | MO2216659 | Malawi, Northern Mzuzu. Pawek 8826 | - | - | - | GU045823 |
| *Conyza gouanii* (L.) Willd. | MO4031623 | Tanzania, Iringa, Ludewa, Livingstone Mtns. Gereau 4047 | - | - | - | AF046948 |
| *Conyza incisa* Aiton | TDNA658 | Mt. Angavokely, Madagascar | EU###### | EU###### | EU###### | EU###### |
| *Conyza limosa* O.Hoffm. | MO2649780 | Malawi, Northern Mzimba. Pawek 11870 | - | - | - | GU045824 |
| *Conyza neocandolleana* Humbert | TDNA675 | Mt. Angavokely, Madagascar | EU###### | EU###### | EU###### | EU###### |
| *Conyza neocandolleana* Humbert | TDNA677 | Mt. Angavokely, Madagascar | EU###### | EU###### | EU###### | EU###### |
| *Conyza neocandolleana* Humbert | TDNA678 | Mt. Angavokely, Madagascar | EU###### | EU###### | EU###### | EU###### |
| *Conyza pinnata* (L. f.) Kuntze | MO3237677 | South Africa, Transvaal, Lothair. Welman 380 | - | - | - | GU045825 |
| *Conyza pyrrhopappa* Sch.Bip. ex A.Rich. | MO3739470 | Tanzania, Tanga, Muheza. Iverson 87419 | - | - | - | AF046953 |
| *Conyza scabrida* DC. | MO4354520 | South Africa, Cape, Voetspadsberg. Van der Kooij s.n. | - | - | - | GU045826 |
| *Conyza stricta* Willd. | MO4031619 | Tanzania, Iringa, Ludewa, Livingstone Mtns. Gereau 3950 | - | - | - | GU045827 |
| *Conyza subscaposa* O.Hoffm. | MO4017065 | Zaire, Massif du Karisimbi. Auquier 2291 | - | - | - | GU045828 |
| *Conyza tigrensis* Oliv. & Hiern | MO4031622 | Tanzania, Mbeya, Mporoto Mtns. Gereau 3770 | - | - | - | GU045829 |
| *Conyza ulmifolia* (Burm. f.) Kuntze | MO3409606 | South Africa, Transvaal, Pretoria. Balsinhas 3526 | - | - | - | GU045830 |
| *Cotula coronopifolia* L. | - | - | - | - | - | AF422118 |
| *Craspedia variabilis* J.Everett & Doust | - | - | - | - | - | EF187684 |
| *Dendranthema grandiflorum* (Ramat.) S.Kitamura | - | - | - | - | - | AF116239 |
| *Ewartia sinclairii* Cheeseman | - | - | - | - | - | U95283 |
| *Felicia aethiopica* (Burm.) Bolus | MO3781368* | South Africa, Cape, Bredasdorp. Rourke 1918 | - | - | - | AF046941 |
| *Felicia namaquana* (Harv.) Merxm. | LHMS1344 | South Africa. JM2895 | - | - | EU###### | EU###### |
| *Gonospermum fruticosum* Less. | - |  | - | - | - | AF155249 |
| *Grangea maderaspatana* (L.) Poir | MO4300678 | Thailand, Chiangmai, Doi Sutep-Pui National Park. Maxwell 90-218 | - | - | - | AF046951 |
| *Helianthus annuus* L. | - | - | - | - | - | AF047927 |
| *Helichrysum lanceolatum* Kirk. | - | - | - | - | - | EU007682 |
| *Leontopodium alpinum* Cass*.* | - | - | - | - | - | FJ639920 |
| *Nidorella resedifolia* DC. | MO4347553 | South Africa, Cape, Britstown. Herman 1060 | - | - | - | AF046952 |
| *Nidorella* sp. Cass. | LHMS2544 | South Africa, Cliffdale Hill, Cliffdale, inland of Durban. Styles 2120 | EU###### | - | - | EU###### |
| *Plecostachys serpyllifolia (Berg.)* Hilliard & B.L.Burtt | - | - | - | - | - | AY445225 |
| *Podocoma notobellidiastrum* (Griseb.) Nesom | MO3713199 | Paraguay, Caazapa, Yuty. Zardini 3009 | - | - | - | AF046963 |
| *Santolina rosmarinifolia* L. | - | - | - | - | - | AJ296387 |
| *Stuartina muelleri* Sond. | - | - | - | - | - | EF187650 |
| *Tagetes patula* L. | - | - | - | - | - | DQ862121 |
