# Supplementary material for: In and out of Madagascar: Dispersal to Peripheral Islands, Insular Speciation and Diversification of Indian Ocean Daisy Trees (Psiadia, Asteraceae)
Source: PLoS One. 2012 Aug 10;7(8):e42932. doi: 10.1371/journal.pone.0042932 (PMC3416790; doi:10.1371/journal.pone.0042932)
Supplement: Table S2 — Primer pairs used in this study. (DOC) [file pone.0042932.s003.doc]

| **Target region** | **Primer** | **Direction** | **Sequence (5´-3´) and length** |
| --- | --- | --- | --- |
| ***acc*D** | 1 | F | AGTATGGGATCCGTAGTAGG (20) |
|  | 4 | R | TCTTTTACCCGCAAATGCAAT (21) |
| ***rpo*B** | 2 | F | ATGCAACGTCAAGCAGTTCC (20) |
|  | 3 | R | CCGTATGTGAAAAGAAGTATA (21) |
| ***psb*A*-trn*H** | psbA | F | GTTATGCATGAACGTAATGCTC (22) |
|  | trnH | R | CGCGCATGGTGGATTCACAAATC (23) |
| **nrITS** | ITS1 | F | TCCGTAGGTGAACCTGCGG (19) |
|  | ITS4 | R | TCCTCCGCTTATTGATATGC (20) |
