# Supplementary material for: In and out of Madagascar: Dispersal to Peripheral Islands, Insular Speciation and Diversification of Indian Ocean Daisy Trees (Psiadia, Asteraceae)
Source: PLoS One. 2012 Aug 10;7(8):e42932. doi: 10.1371/journal.pone.0042932 (PMC3416790; doi:10.1371/journal.pone.0042932)
Supplement: Table S3 — DNA isolation, purification and PCR conditions. Notes: Total DNA was isolated using the BioSprint DNA Plant Kit (Qiagen, Valencia, CA) with a modified protocol, using ball bearings and silica powder. All markers were amplified from genomic DNA using PCR. Each 25 µl reaction contained 0.1 µl (5.0 units) of GoTaq polymerase (Promega, Madison, WI), 5 µl of 5x reaction buffer, 0.5 µl dNTP (10 mM stock solution), 0.5 µl of each primer (10mM stock solution) and 1.0 µl template DNA. PCR products were cleaned using the MinElute PCR Purification Kit (Qiagen, Valencia, CA). Sequencing was performed on an ABI 3730xl (Genoscope, Evry, France). (DOC) [file pone.0042932.s004.doc]

| ***acc*D and *rpo*B** | **Length (min:sec)** | **Temperature (°C)** | **Number of cycles** |
| --- | --- | --- | --- |
| ***Initial denature*** | 1:00 | 94 |  |
| ***Denature*** | 0:30 | 94 | 38x |
| ***Annealing*** | 0:40 | 53 |  |
| ***Extension*** | 0:40 | 72 |  |
| ***Final extension*** | 5:00 | 72 |  |

| ***psb*A-*trn*H** | **Length (min:sec)** | **Temperature (°C)** | **Number of cycles** |
| --- | --- | --- | --- |
| ***Initial denature*** | 5:00 | 94 |  |
| ***Denature*** | 1:00 | 94 | 28x |
| ***Annealing*** | 1:00 | 50 |  |
| ***Extension*** | 2:00 | 72 |  |
| ***Final extension*** | 10:00 | 72 |  |

| **nrITS** | **Length (min:sec)** | **Temperature (°C)** | **Number of cycles** |
| --- | --- | --- | --- |
| ***Initial denature*** | 2:00 | 96 |  |
| ***Denature*** | 0:30 | 94 | 40x |
| ***Annealing*** | 0:30 | 53 |  |
| ***Extension*** | 2:00 | 75 |  |
| ***Final extension*** | 7:00 | 75 |  |
