# Supplementary material for: In and out of Madagascar: Dispersal to Peripheral Islands, Insular Speciation and Diversification of Indian Ocean Daisy Trees (Psiadia, Asteraceae)
Source: PLoS One. 2012 Aug 10;7(8):e42932. doi: 10.1371/journal.pone.0042932 (PMC3416790; doi:10.1371/journal.pone.0042932)
Supplement: Table S4 — Calibration scheme and results of molecular dating analyses for selected nodes using Penalized Likelihood and BEAST. Notes: Values in millions of years ago. Abbreviations: HPD – Highest Posterior Density interval; (logN) – logNormal prior; (N) – Normal prior; PL – Penalized Likelihood; PP – Posterior Probability; UCLN – uncorrelated lognormal; (U) – Uniform prior. (DOC) [file pone.0042932.s005.doc]

| **Dating methods** |  | **Penalized Likelihood 1** |  |  | **Penalized Likelihood 2** |  |  |
| --- | --- | --- | --- | --- | --- | --- | --- |
|  | **Constraints** | **Penalty = additive** |  |  | **Penalty = additive** |  |  |
| **Priors** | **Asteroideae crown age (root)** | fix = 32.5 |  |  | fix = 32.5 |  |  |
|  | ***Tagetes*-*Helianthus* split** | - |  |  | min = 16.9 |  |  |
|  | **Gnaphalieae node** | - |  |  | min = 12.0 |  |  |
|  | ***Artemisia* root age** | - |  |  | min = 5.3 |  |  |
|  | **Nodes of interest PL** | **Mean** | **Min** | **Max** | **Mean** | **Min** | **Max** |
| **Posteriors** | **Asteroideae crown age (root)** | 32.5 | - | - | 32.5 | - | - |
|  | ***Tagetes*-*Helianthus* split** | 22.03 | 12.74 | 32.5 | 22.26 | 16.9 | 32.5 |
|  | **Gnaphalieae node** | 8.65 | 5.13 | 21.18 | 20.63 | 20.6 | 30.26 |
|  | ***Artemisia* root age** | 7.88 | 3.09 | 30.17 | 7.98 | 5.3 | 25.24 |
|  | **Clade B root age** | 7.57 | 1.54 | 18.74 | 7.98 | 1.54 | 18.86 |
|  | **Clade B crown age** | 6.61 | 1.2 | 15.44 | 7.03 | 1.19 | 16.07 |
|  | **Clade A root age** | 14.48 | 5.64 | 23.13 | 15.05 | 5.65 | 31.64 |
|  | **Clade A crown age** | 12.98 | 3.83 | 22.4 | 13.5 | 3.86 | 31.57 |
|  | **Clade A *Psiadia* Mascarenes root age** | 9.45 | 0.81 | 18.9 | 9.97 | 1.63 | 30.88 |
|  | **Clade A *Psiadia* Mascarenes crown age** | 5.46 | 0.43 | 17.03 | 5.88 | 0.58 | 15.53 |
| **Dating methods (continued)** |  | **BEAST 1 (UCLN)** |  |  | **BEAST 2 (UCLN)** |  |  |
|  | **Constraints** |  |  |  |  |  |  |
| **Priors (type)** | **Asteroideae crown age (root)** | (U) 14.1-100 |  |  | (N) 32.5 (14.1-50.9) |  |  |
|  | ***Tagetes*-*Helianthus* split** | (U) 16.9-44.1 |  |  | (logN) 22.3 (16.9-44.1) |  |  |
|  | **Gnaphalieae node** | (U) 12.0-29.3 |  |  | (N) 20.0 (12.0-29.3) |  |  |
|  | ***Artemisia* root age** | (U) 5.3-33.9 |  |  | (logN) 16.98 (5.44-28.52) |  |  |
|  | **Nodes of interest BEAST** | **Age estimate** | **95% HPD** | **PP** | **Age estimate** | **95% HPD** | **PP** |
| **Posteriors** | **Asteroideae crown age (root)** | 55.93 | (31.65-86.07) | 1 | 39.4 | (28.5-51.03) | 1 |
|  | ***Tagetes*-*Helianthus* split** | 26.88 | (16.9-40.04) | 1 | 20.7 | (18.12-23.82) | 1 |
|  | **Gnaphalieae node** | 17.14 | (12.0-25.52) | 1 | 14.69 | (8.67-20.48) | 1 |
|  | ***Artemisia* root age** | 12.45 | (5.3-21.51) | 1 | 8.48 | (5.81-12.19) | 1 |
|  | **Clade B root age** | 6.8 | (3.01-11.45) | 1 | 5.11 | (2.56-7.8) | 1 |
|  | **Clade B crown age** | 5.75 | (2.52-9.73) | 0.92 | 4.32 | (2.25-6.69) | 0.92 |
|  | **Clade A root age** | 13.53 | (6.68-21.32) | 0.52 | 10.08 | (5.86-14.48) | 0.52 |
|  | **Clade A crown age** | 11.11 | (5.59-18.14) | 0.98 | 8.21 | (4.76-12.15) | 0.98 |
|  | **Clade A *Psiadia* Mascarenes root age** | 4.7 | (2.12-7.99) | 1 | 3.51 | (1.76-5.58) | 1 |
|  | **Clade A *Psiadia* Mascarenes crown age** | 3.13 | (1.3-5.45) | 1 | 2.33 | (1.1-3.83) | 1 |
