# Supplementary material for: In and out of Madagascar: Dispersal to Peripheral Islands, Insular Speciation and Diversification of Indian Ocean Daisy Trees (Psiadia, Asteraceae)
Source: PLoS One. 2012 Aug 10;7(8):e42932. doi: 10.1371/journal.pone.0042932 (PMC3416790; doi:10.1371/journal.pone.0042932)
Supplement: Table S5 — Inferred ancestral ranges for branches (separated by vertical bar) descending from each node in Figure 2 , and their relative probability. Note: Only alternative scenarios that fall within two log-likelihood units of the optimal reconstruction and have a relative probability ≥0.1 are provided. Asterisks highlight nodes for which multiple equally probable solutions were retrieved using Lagrange and/or S-DIVA. Clades for which differences exist in optimal reconstructions between Lagrange and S-DIVA are shown in bold. (DOC) [file pone.0042932.s006.doc]

| **Clade no.** | **Infer. anc. area (Lagrange)** | **Relative probability (Lagrange)** | **Mean age estimate [95%HPD] (in Ma) (BEAST)** | **Clade support (BEAST)** | **Infer. anc. area (S-DIVA)** | **Marginal probability (S-DIVA)** |
| --- | --- | --- | --- | --- | --- | --- |
| **1*** | **[D|BD]** | **0.23** | **16.23 [9.93,23.45]** | **PP=1.00** | **AD** | **26.95** |
|  | **[D|AD]** | **0.23** |  |  | **BD** | **26.13** |
|  | **[D|D]** | **0.12** |  |  | **D** | **26.02** |
|  |  |  |  |  | DF | 20.9 |
| 2* | [B|D] | 0.24 | 13.92 [8.35,20.01] | PP=0.97 | AD | 31.17 |
|  | [A|D] | 0.24 |  |  | BD | 26.44 |
|  |  |  |  |  | AF | 23.18 |
|  |  |  |  |  | BF | 19.21 |
| 3 | [D|D] | 0.35 | 11.11 [6.76,16.13] | PP=1.00 | D | 65.33 |
|  | [D|DF] | 0.22 |  |  | F | 34.67 |
|  | [F|DF] | 0.17 |  |  |  |  |
|  | [F|F] | 0.12 |  |  |  |  |
| 4 | [F|D] | 0.68 | 10.08 [5.86,14.48] | PP=0.52 | DF | 77.46 |
|  |  |  |  |  | EF | 22.54 |
| 5 | [E|D] | 0.41 | 8.29 [4.34,12.57] | PP=0.80 | DE | 100 |
|  | [D|D] | 0.38 |  |  |  |  |
|  | [F|D] | 0.11 |  |  |  |  |
| 6 | [D|D] | 0.96 | 6.97 [3.14,11.27] | PP=0.38 | D | 100 |
| 7 | [F|F] | 0.92 | 8.21 [4.76,12.15] | PP=0.97 | F | 100 |
| 8 | [F|F] | 0.95 | 6.77 [3.73,10.02] | PP=1.00 | F | 100 |
| 9 | [F|F] | 0.84 | 5.38 [2.96,8.14] | PP=1.00 | F | 100 |
| 10 | [F|F] | 0.98 | 4.58 [2.39,7.17] | PP=0.63 | F | 100 |
| 11 | [F|F] | 0.89 | 3.73 [1.8,6.01] | PP=0.98 | F | 100 |
|  | [CF|F] | 0.11 |  |  |  |  |
| 12 | [F|F] | 1.00 | 2.46 [0.87,4.27] | PP=0.85 | F | 100 |
| 13 | [F|C] | 0.89 | 2.3 [0.59,4.21] | PP=0.49 | CF | 100 |
| 14 | [F|F] | 1.00 | 0.9 [0.03,2.31] | PP=1.00 | F | 100 |
| 15 | [F|J] | 0.48 | 3.51 [1.76,5.58] | PP=1.00 | FJ | 75.05 |
|  | [F|I] | 0.46 |  |  | FI | 24.95 |
| 16 | [I|J] | 0.91 | 2.33 [1.1,3.83] | PP=1.00 | IJ | 100 |
| 17 | [J|J] | 0.98 | 1.68 [0.43,2.93] | PP=0.42 | J | 100 |
| 18 | [I|I] | 0.91 | 1.69 [0.72,2.72] | PP=0.91 | I | 100 |
| 19 | [I|I] | 1.00 | 1.12 [0.39,1.98] | PP=0.95 | I | 100 |
| 20 | [I|I] | 1.00 | 0.5 [0.05,1.09] | PP=0.98 | I | 100 |
| 21 | [I|I] | 1.00 | 0.19 [0,0.56] | PP=0.56 | I | 100 |
| 22 | [I|I] | 0.79 | 1.35 [0.53,2.25] | PP=0.34 | I | 100 |
|  | [HI|I] | 0.21 |  |  |  |  |
| 23 | [I|I] | 1.00 | 0.35 [0.01,0.89] | PP=1.00 | I | 100 |
| 24 | [I|H] | 0.96 | 1 [0.28,1.86] | PP=0.30 | HI | 100 |
| 25 | [H|H] | 0.99 | 0.36 [0,1.06] | PP=0.65 | H | 100 |
| 26 | [I|I] | 1.00 | 0.55 [0.04,1.27] | PP=0.97 | I | 100 |
| 27 | [F|F] | 0.99 | 1.95 [0.65,3.57] | PP=0.94 | F | 100 |
| 28 | [F|F] | 1.00 | 0.74 [0.08,1.68] | PP=0.97 | F | 100 |
| 29 | [F|F] | 0.98 | 1.3 [0.32,2.57] | PP=0.22 | F | 100 |
| 30 | [F|F] | 0.94 | 0.84 [0.12,1.81] | PP=0.22 | F | 100 |
| 31 | [F|F] | 0.74 | 0.5 [0.01,1.25] | PP=0.25 | F | 100 |
|  | [DF|F] | 0.25 |  |  |  |  |
| 32 | [D|F] | 0.99 | 0.29 [0,0.9] | PP=0.23 | DF | 100 |
| 33 | [F|F] | 0.99 | 3.34 [1.21,5.99] | PP=1.00 | F | 100 |
| 34 | [F|F] | 1.00 | 2.18 [0.66,4.04] | PP=1.00 | F | 100 |
| 35 | [F|F] | 1.00 | 0.27 [0,0.86] | PP=1.00 | F | 100 |
| 36 | [F|F] | 1.00 | 1.68 [0.41,3.22] | PP=0.31 | F | 100 |
| 37 | [D|D] | 0.40 | 7.26 [3.79,11.19] | PP=1.00 | D | 48.9 |
|  | [F|F] | 0.19 |  |  | F | 37.55 |
|  | [DF|D] | 0.12 |  |  | DF | 13.55 |
|  | [F|DF] | 0.11 |  |  |  |  |
| **38** | **[DF|D]** | **0.53** | **2.82 [0.64,5.54]** | **PP=1.00** | **D** | **57.05** |
|  | **[D|D]** | **0.46** |  |  | **DF** | **42.95** |

| **Clade no.** | **Infer. anc. area (Lagrange)** | **Relative probability (Lagrange)** | **Mean age estimate [95%HPD] (in Ma) (BEAST)** | **Clade support (BEAST)** | **Infer. anc. area (S-DIVA)** | **Marginal probability (S-DIVA)** |
| --- | --- | --- | --- | --- | --- | --- |
| 39* | [D|D] | 0.35 | 6.56 [3.38,10.29] | PP=0.15 | DF | 33.33 |
|  | [F|F] | 0.29 |  |  | F | 33.33 |
|  |  |  |  |  | D | 33.33 |
| **40*** | **[CDF|F]** | **0.34** | **2.54 [0.64,4.89]** | **PP=1.00** | **DF** | **50** |
|  | **[DF|F]** | **0.33** |  |  | **F** | **50** |
|  | **[F|F]** | **0.22** |  |  |  |  |
| 41 | [B|A] | 0.22 | 11.26 [5.66,17.71] | PP=0.52 | AB | 100 |
|  | [B|B] | 0.16 |  |  |  |  |
|  | [A|A] | 0.16 |  |  |  |  |
| 42 | [D|D] | 0.71 | 12.27 [6.95,18.33] | PP=1.00 | D | 100 |
|  | [D|DF] | 0.24 |  |  |  |  |
| 43 | [D|D] | 0.46 | 8.92 [4.66,13.42] | PP=1.00 | D | 53.6 |
|  | [D|DF] | 0.36 |  |  | DF | 46.4 |
|  | [D|F] | 0.12 |  |  |  |  |
| **44** | **[F|DF]** | **0.75** | **7.35 [4,11.33]** | **PP=0.96** | **DH** | **38.76** |
|  | **[F|F]** | **0.12** |  |  | **F** | **30.62** |
|  |  |  |  |  | **DF** | **30.62** |
| 45 | [F|H] | 0.91 | 5.11 [2.56,7.8] | PP=1.00 | FH | 100 |
| 46 | [H|H] | 0.98 | 4.32 [2.25,6.69] | PP=0.92 | H | 100 |
| 47 | [H|H] | 1.00 | 1.78 [0.55,3.34] | PP=1.00 | H | 100 |
| 48 | [H|H] | 1.00 | 1.05 [0.2,2.13] | PP=0.91 | H | 100 |
| 49 | [H|H] | 1.00 | 0.2 [0,0.62] | PP=1.00 | H | 100 |
| 50 | [H|H] | 1.00 | 0.23 [0,0.72] | PP=0.99 | H | 100 |
| 51 | [H|H] | 1.00 | 3.14 [1.46,4.98] | PP=1.00 | H | 100 |
| 52 | [H|H] | 1.00 | 1.77 [0.69,3.06] | PP=1.00 | H | 100 |
| 53 | [H|H] | 1.00 | 1.27 [0.42,2.29] | PP=0.79 | H | 100 |
| 54 | [H|H] | 1.00 | 0.65 [0.11,1.35] | PP=1.00 | H | 100 |
| 55 | [H|H] | 1.00 | 0.7 [0.01,1.72] | PP=0.79 | H | 100 |
| 56 | [H|H] | 1.00 | 2.59 [1.12,4.27] | PP=0.33 | H | 100 |
| 57 | [H|H] | 1.00 | 0.49 [0,1.29] | PP=1.00 | H | 100 |
| 58 | [H|H] | 1.00 | 2.28 [0.82,3.92] | PP=0.26 | H | 100 |
| 59 | [F|F] | 1.00 | 0.69 [0.05,1.77] | PP=1.00 | F | 100 |
| 60 | [F|F] | 1.00 | 0.24 [0,0.72] | PP=0.57 | F | 100 |
| 61 | [D|D] | 0.99 | 2.54 [0.31,5.53] | PP=1.00 | D | 100 |
| 62 | [D|D] | 0.97 | 10.51 [5.3,15.88] | PP=0.54 | D | 100 |
| 63 | [D|D] | 0.99 | 6.54 [2.23,11.46] | PP=0.97 | D | 100 |
| 64 | [D|D] | 1.00 | 1.51 [0.13,3.59] | PP=1.00 | D | 100 |
| 65 | [D|D] | 0.94 | 9.23 [4.26,14.58] | PP=0.45 | D | 100 |
